# Supplementary material for: Precision tumor-on-chip for personalized assessment of drug efficacy and immune cell delivery
Source: Cell Rep Methods. 2026 Jun 4;6(8):101475. doi: 10.1016/j.crmeth.2026.101475 (PMC13494545; doi:10.1016/j.crmeth.2026.101475)
Supplement: Supplementary file 1 [file mmc1.pdf]

Cell Reports Methods, Volume *6*

Supplemental information

Precision tumor-on-chip for personalized assessment of drug efficacy and immune cell delivery

Elena Kremneva, Johannes Smolander, Mimosa Peltokangas, Mikaela Grönholm, Lilja Lahtinen, Shadi Jansouz, Sanna Vainionpää, Bassel Alsaed, Kristian Borenius, Anniina Palojärvi, Pauliina Junttila, Jere Kettunen, Tuomas Pylkkö, Michaela Feodoroff, Gabriella Antignani, Harri K. Mustonen, Markus Vähä-Koskela, Tuan Nguyen, Prateek Singh, Toni T. Seppälä, Eva Sutinen, Reetta Riikonen, Satu Juhila, Vincenzo Cerullo, Hanna Seppänen, Ilkka Ilonen, Sebastien Mosser, and Heidi M. Haikala

DOCUMENT S1

Figures S1–S3

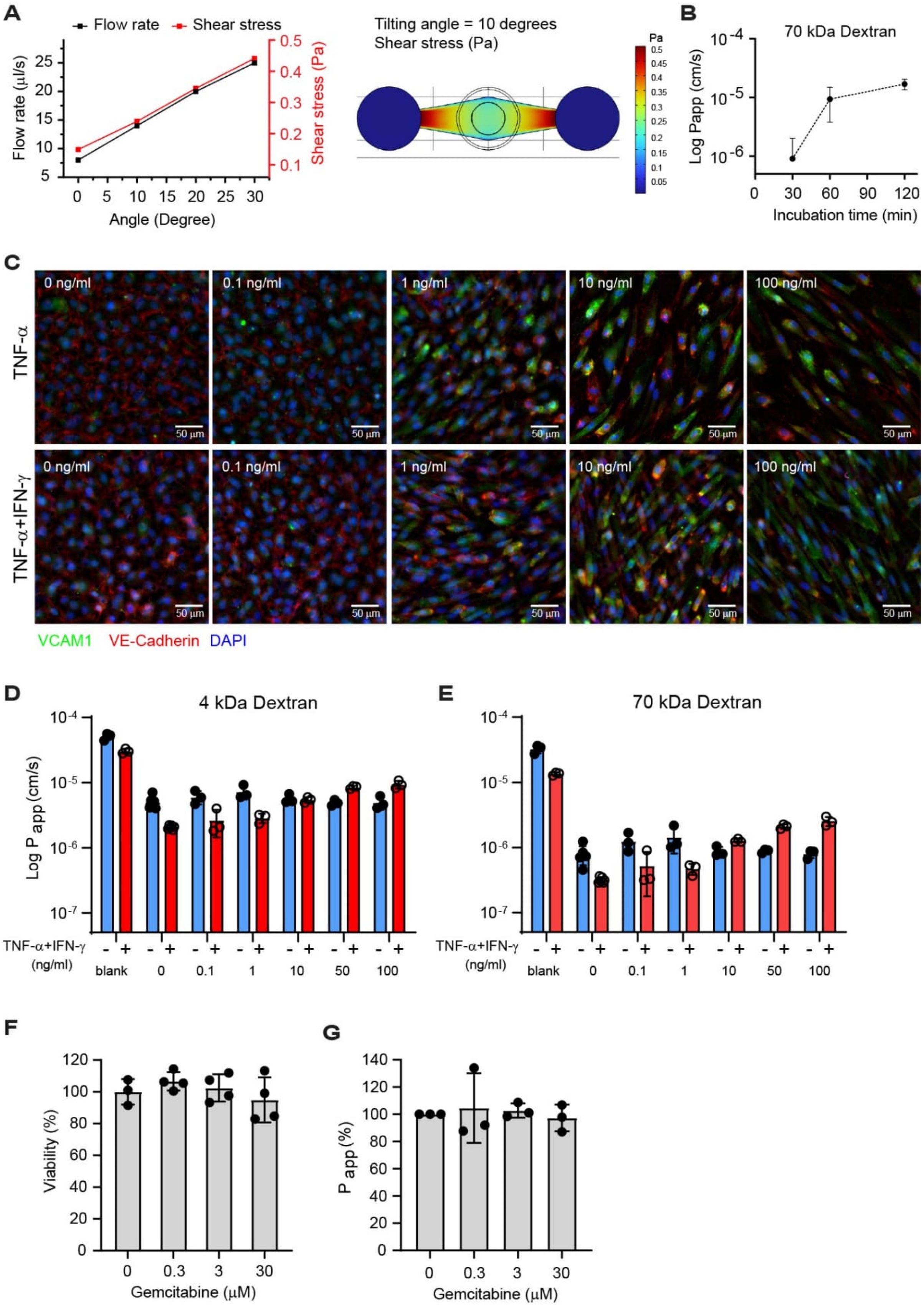
A
Flow rate
Shear stress
Flow rate (µl/s)
Shear stress (Pa)
Angle (Degree)
Tilting angle = 10 degrees
Shear stress (Pa)
Pa
B
70 kDa Dextran
Log Papp (cm/s)
Incubation time (min)
C
TNF-α
TNF-α+IFN-γ
0 ng/ml
0.1 ng/ml
1 ng/ml
10 ng/ml
100 ng/ml
50 µm
VCAM1 VE-Cadherin DAPI
D
4 kDa Dextran
Log P app (cm/s)
TNF-α+IFN-γ (ng/ml)
blank 0 0.1 1 10 50 100
E
70 kDa Dextran
TNF-α+IFN-γ (ng/ml)
blank 0 0.1 1 10 50 100
F
Viability (%)
Gemcitabine (µM)
G
P app (%)
Gemcitabine (µM)

Figure S1. Response of endothelial barrier made of HUVEC cells to (TNF-α+IFN-g)-induced inflammation. Related to Figure 1. (A) Modeling of flow rate and shear stress changes depending on angle set on the rocker and spatial distribution of shear stress values at 10-degree angle. Simulation was done using finite element analysis in COMSOL Multiphysics. **(B)** Assessment of Dextran permeability with time through bare membrane. Measurements were done at 30, 60 and 120 min, and permeability was plotted in log scale for better visualization. **(C)** VCAM1 (green) and VE-cadherin (red) staining of HUVEC endothelial barrier. Cells were treated with 0-100 ng/mL TNF-α alone or TNF-α+IFN-γ mixture for 48 hours, imaged, and analyzed for expression levels. DAPI was used as a nuclear stain. Scale bar = 50 µm. **(D, E)** Permeability of HUVEC endothelial monolayers in microfluidic channels before and after (TNF-α+IFN-γ)-induced inflammation. Cells were incubated with TNF-α and IFN-γ at concentrations from 0 to 100 ng/mL for 24 hours, and permeability was assessed using 4 kDa (B) and 70 kDa (C) dextran. Sample size = 3 for each condition, data shown as mean ± SD. **(F, G)** Viability of endothelial cells (HUVECs) cultured in microfluidic channel (E) and permeability (P_{app} in %) of endothelial barrier (F) after 3-day treatment with Gemcitabine at varying concentrations. Cell viability was calculated based on a fluorescence signal from live and dead cells and presented as live/dead ratio normalized to control condition without added drug. Barrier permeability was assessed using 70 kDa Dextran and shown in relation to permeability in the absence of the drug. Data shown as mean ± SD (3-4 replicas).

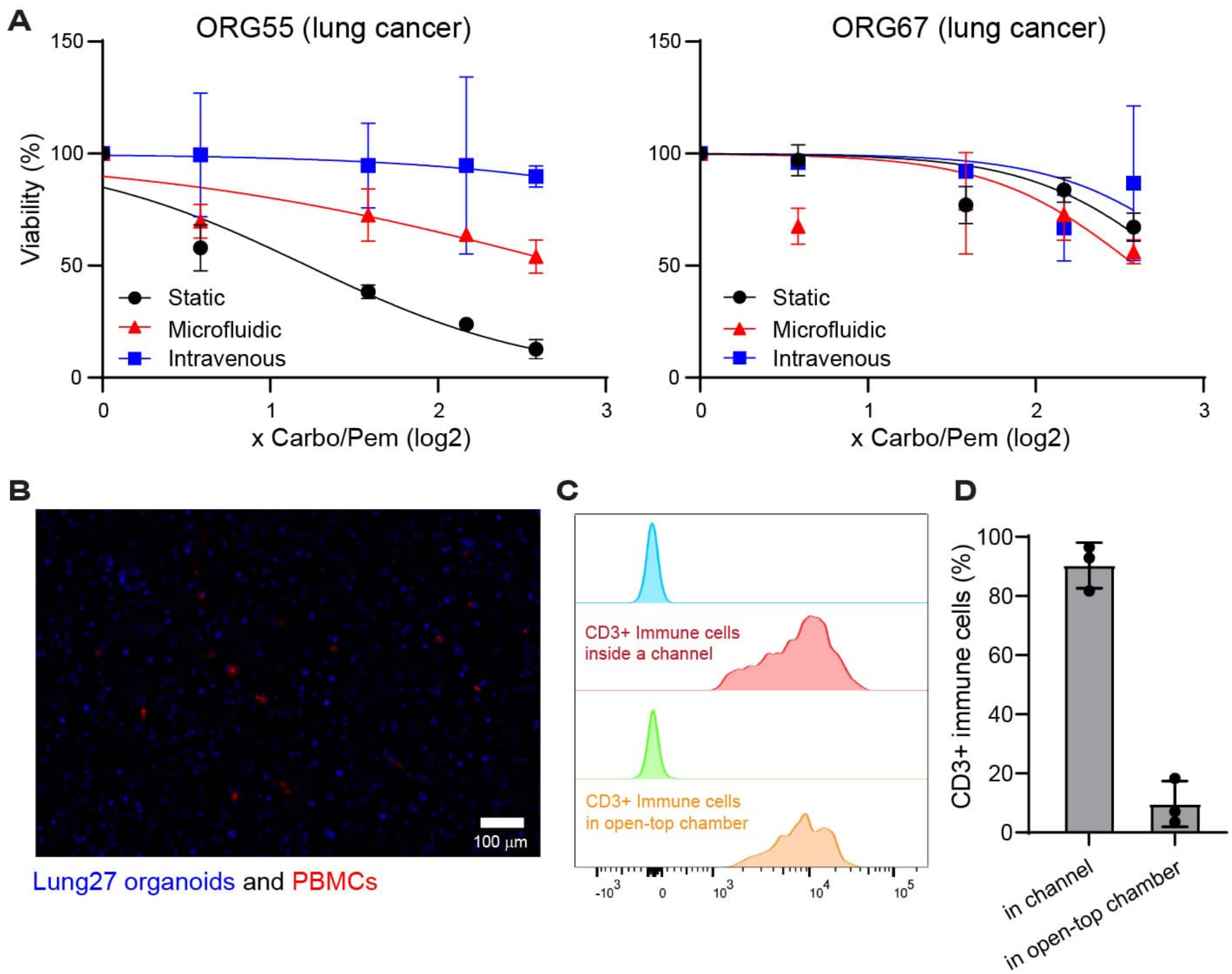

Figure S2. Assessment of drug responses and immune cell infiltration in microfluidic TOC. Related to Figures 2 and 3. (A) IC50 drug response curves for ORG55 and ORG67 cultured in a 384-well plate ("Static") or open-top chambers of TOC ("Microfluidic"). Chemotherapeutics (Carboplatin/Pemetrexed) were added to cells in either setup, and viability was assessed using CellTiter-Glo Luminescent Cell Viability assay. **(B)** Fluorescence image of open-top chamber with Lung27 organoids (blue) and infiltrated PBMCs (red). Organoids and PBMCs were pre-labeled with live stains. Magnification 10x, scale bar = 100 µm. **(C, D)** Quantification of infiltrated PBMCs in the absence of virus infection using flow cytometry. Non-infected organoids and PBMCs were stained with EpCam and CD3 antibodies, respectively. Only CD3+ lymphocytes were quantified. The average percentage of cells in each compartment is shown as mean ± SD (n=3).

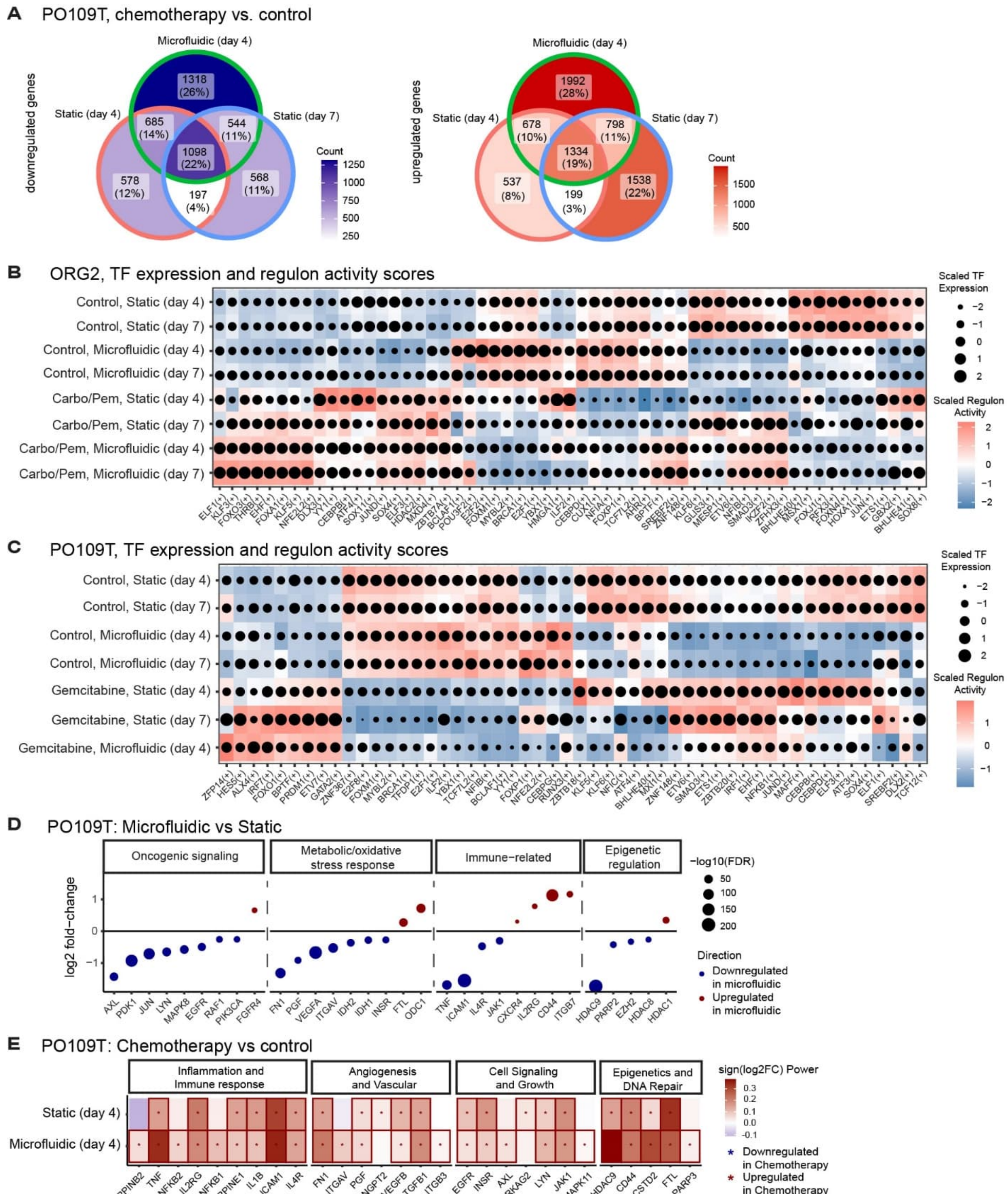

Figure S3. Effect of cancer chemotherapeutics on overall gene and transcriptional factor expression activity. Related to Figures 6 and 7. (A) Schematic showing total gene numbers (common and unique) upregulated (red scale) or downregulated (blue scale) in Microfluidic vs Static conditions, with or without drug treatment, for PO109T cancer models. **(B, C)** Comparison of transcription factor (TF) expression and regulon activity scores for ORG2 (B) and PO109T (C) models under different culturing conditions and drug treatments. Color coding indicates increased (red) or decreased (blue) regulon activity, with black circle

size representing TF expression intensity. No data is shown for PO109T on day 7 in microfluidic settings due to low cell recovery. (**D**) Differential expression of genes in oncogenic, metabolic/oxidative stress response, immune-related, and epigenetic regulation pathways between Microfluidic and Static conditions for PO109T. Red and blue dots represent upregulated and downregulated genes in Microfluidic conditions, respectively, with spot size indicating FDR. (**E**) Chemotherapeutic treatments cause different gene expression changes in Static vs Microfluidic conditions for PO109T, with significant differences highlighted in square frames.
