# Supplementary material for: Precision tumor-on-chip for personalized assessment of drug efficacy and immune cell delivery
Source: Cell Rep Methods. 2026 Jun 4;6(8):101475. doi: 10.1016/j.crmeth.2026.101475 (PMC13494545; doi:10.1016/j.crmeth.2026.101475)
Supplement: Supplementary file 2 [file mmc4.pdf]

Cell Reports
Methods

Article

Precision tumor-on-chip for personalized assessment of drug efficacy and immune cell delivery

Graphical abstract

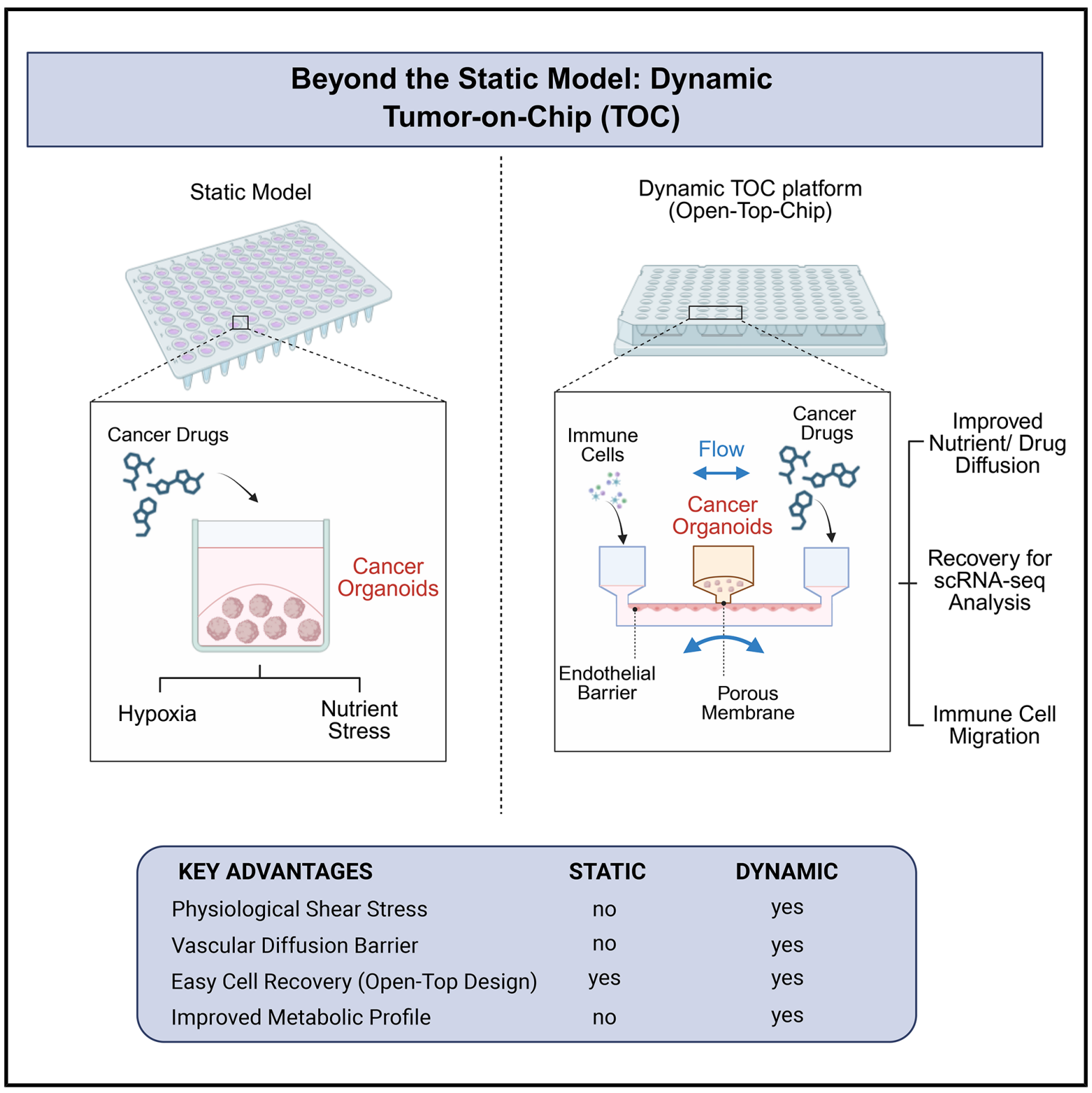

Authors

Elena Kremneva, Johannes Smolander, Mimosa Peltokangas, ..., Ilkka Ilonen, Sebastien Mosser, Heidi M. Haikala

Correspondence

heidi.haikala@helsinki.fi

In brief

Kremneva et al. present a microfluidic tumor-on-chip platform integrating patient-derived organoids with vascular flow to simulate physiological drug delivery. This model captures treatment resistance features at single-cell resolution and models immune cell migration. By linking transport dynamics with therapeutic efficacy, the platform provides a scalable tool for personalized drug evaluation.

Highlights

- Fluidic system integrates patient-derived organoids with endothelial and immune cells
- Controlled fluid flow recreates physiologically constrained drug delivery dynamics
- The TOC captures cellular features of treatment resistance at single-cell resolution
- The platform enables analysis of tumor-specific immune behavior across multiple cancers

Kremneva et al., 2026, Cell Reports Methods 6, 101475
August 17, 2026 © 2026 The Authors. Published by Elsevier Inc.
https://doi.org/10.1016/j.crmeth.2026.101475

CellPress

Cell Reports Methods

CellPress
OPEN ACCESS

Article

Precision tumor-on-chip for personalized assessment of drug efficacy and immune cell delivery

Elena Kremneva,[1,2,3] Johannes Smolander,[1,2,3,4] Mimosa Peltokangas,[5] Mikaela Grönholm,[1,2,6,7] Lilja Lahtinen,[1,2,3] Shadi Jansouz,[1,2,3] Sanna Vainionpää,[8] Bassel Alsaed,[1,2,3] Kristian Borenius,[2,9] Anniina Palojärvi,[5] Pauliina Junttila,[5] Jere Kettunen,[5] Tuomas Pylkkö,[10] Michaela Feodoroff,[1,2,3,6,7] Gabriella Antignani,[1,2,6,7] Harri K. Mustonen,[2,11,12] Markus Vähä-Koskela,[2,11] Tuan Nguyen,[5] Prateek Singh,[5] Toni T. Seppälä,[2,4,8,13,14,15] Eva Sutinen,[2,16] Reetta Riikonen,[17] Satu Juhila,[18] Vincenzo Cerullo,[1,2,6,7,19] Hanna Seppänen,[2,11,12] Ilkka Ilonen,[2,9] Sebastien Mosser,[5] and Heidi M. Haikala[1,2,3,4,20,21,*]

[1]Translational Immunology Research Program (TRIMM), Research Programs Unit, Faculty of Medicine, University of Helsinki, Helsinki, Finland
[2]iCAN Digital Precision Cancer Medicine Flagship, Helsinki, Finland
[3]Institute for Molecular Medicine Finland (FIMM), Helsinki Institute for Life Sciences (HiLIFE), University of Helsinki, Helsinki, Finland
[4]Center for Cancer Eradication Research (CCER), Faculty of Medicine and Health Technology, Tampere University, Tampere, Finland
[5]Finnadvance Oy, 00790 Helsinki, Finland
[6]Laboratory of Immunovirotherapy, Drug Research Program, Faculty of Pharmacy, University of Helsinki, Helsinki, Finland
[7]Drug Delivery, Drug Research Program, Division of Pharmaceutical Biosciences, Faculty of Pharmacy, University of Helsinki, Helsinki, Finland
[8]Department of Abdominal Surgery, Helsinki University Hospital and University of Helsinki, Helsinki, Finland
[9]Department of General Thoracic and Esophageal Surgery, Heart and Lung Center, Helsinki University Hospital & University of Helsinki, Helsinki, Finland
[10]Drug Research Program, Faculty of Pharmacy, University of Helsinki, Helsinki, Finland
[11]Translational Cancer Medicine Program, University of Helsinki and Helsinki University Hospital (HUS), Helsinki, Finland
[12]Department of Gastrointestinal Surgery, Helsinki University Hospital & University of Helsinki, Helsinki, Finland
[13]Applied Tumor Genomics Research Program, Research Programs Unit, University of Helsinki, Helsinki, Finland
[14]Department of Gastroenterology and Alimentary Tract Surgery, Tampere University Hospital, Tampere, Finland
[15]TAYS Cancer Centre, Tampere University and Tampere University Hospital, Tampere, Finland
[16]Department of Pulmonary Medicine, Heart and Lung Center, Helsinki University Hospital & University of Helsinki, Helsinki, Finland
[17]Orion Pharma, Oncology Research, 20360 Turku, Finland
[18]Orion Pharma, Oncology Development, 02200 Espoo, Finland
[19]Department of Molecular Medicine and Medical Biotechnology and CEINGE, Naples University Federico II, Naples, Italy
[20]Research unit, Heart and Lung Center, Helsinki University Hospital, Helsinki, Finland
[21]Lead contact
*Correspondence: heidi.haikala@helsinki.fi
https://doi.org/10.1016/j.crmeth.2026.101475

MOTIVATION Tumor organoid models enable patient-specific drug testing but typically expose cells to uniform drug concentrations and lack vascular delivery dynamics, limiting their physiological relevance. As a result, it remains difficult to assess how drug transport and microenvironmental interactions influence therapeutic responses. To address this gap, we developed a microfluidic tumor-on-chip (TOC) platform that integrates patient-derived organoids with controlled fluid flow and an optional endothelial barrier to simulate vascular drug delivery. This system enables analysis of drug efficacy, delivery-constrained dose-response behavior, and tumor-induced immune cell migration, providing a methodological framework that links transport processes with patient-specific therapeutic responses.

SUMMARY

Tumor heterogeneity and drug resistance limit the efficacy of cancer therapies and highlight the need for patient-specific preclinical models. Here, we develop a microfluidic tumor-on-chip (TOC) platform that integrates patient-derived organoids with a functional endothelial barrier and utilizes liquid flow to recreate drug delivery from the vasculature into tumor tissue. This system enables evaluation of therapeutic efficacy and toxicity under physiologically relevant conditions. Additionally, the platform is utilized to assess immune

© 2026 The Authors. Published by Elsevier Inc.
This is an open access article under the CC BY license (http://creativecommons.org/licenses/by/4.0/).

cell migration induced by tumor-derived factors in the absence of an endothelial barrier. In pancreatic cancer, the TOC model captures cellular features associated with treatment resistance and pathway rewiring at single-cell resolution, with drug response patterns showing a trend toward alignment with patient clinical outcomes in a limited, retrospective, exploratory setting. By simulating vascular drug transport, the platform provides a scalable and clinically relevant tool for studying treatment response mechanisms and evaluating drug responses under physiologically constrained delivery conditions.

INTRODUCTION

Novel cancer therapies show promise, yet challenges in effectively targeting the disease persist. Tumor heterogeneity, driven by epigenetic events and continuous mutagenesis, results in variable responses to therapies, complicating treatment efficacy and prognostication.[1] Furthermore, difficulties in early detection and selecting optimal treatments continue to impede successful outcomes. Current diagnostic methods and treatment protocols often fail to fully account for individual variability in tumor biology and patient responses, highlighting the critical need for reliable and personalized *ex vivo* models that can more precisely predict individual treatment responses and help clinicians select the optimal treatment course.

Current preclinical models in cancer research struggle to capture the complexity and heterogeneity of human tumors.[2] For example, while two-dimensional (2D) cell lines offer simplicity and ease of use, they lack extracellular matrix and fail to replicate tumors' three-dimensional (3D) structure and *in vivo* heterogeneity. This lack of complexity has led to oversimplified views of tumor biology that may not accurately reflect clinical scenarios. Moreover, significant differences in gene expression patterns between 2D cell lines and their *in vivo* counterparts have been documented.[3] Patient-derived xenograft (PDX) models, which involve grafting patient tumors into mice, retain cellular heterogeneity but replace the original tumor microenvironment with murine cells, resulting in treatment response deviations from the original tumor. Additionally, mouse models are labor-intensive and costly.[4,5]

Patient-derived tumor organoids, 3D structures cultured from tumor tissue, better preserve tumor heterogeneity than traditional cancer models.[6] Integrating tissues, cell cultures, or organoids with microfluidic technologies has led to the development of organ-on-chip (OOC) systems.[7] These systems leverage cell biology and engineering to recapitulate aspects of *in vivo* complexity, helping to bridging the gap between preclinical testing and clinical applications. Recent OOC platforms have successfully mimicked functions of major human organs, such as the lung, liver, heart, and brain, and are currently used primarily for toxicity testing.[8] In OOC systems, the presence of isolated microchannels and the ability to induce shear forces, like those experienced by endothelial cells in the bloodstream, enable the formation of vasculature-like structures and the study of tissue-vasculature interfaces, which are critical for studying drug permeability and efficacy.[9,10]

Tumor-on-chip (TOC) platforms have been created to study cancer growth and metastasis using cell lines or organoids.[11,12] The main advantage of TOC systems is that they provide a more physiologically relevant setup for studying disease mechanisms, and drug responses compared to conventional 2D cell cultures and animal models, aiming at higher predictability of patient outcome. However, most TOCs remain at the proof-of-concept stage. Many of these systems have complex designs that, while enabling the study of intricate cellular interactions, often require complicated peripheral equipment or costly disposable accessories.[8,11] Additionally, most platforms do not allow for the retrieval of cells for further analysis, limiting the ability to study the underlying cellular mechanisms of observed phenotypes.[11,12]

In this study, we used an open-top microfluidic platform to develop a TOC system that can more accurately replicate patient-specific tumor heterogeneity and drug distribution across physical barriers, while maintaining a scalable throughput. We compared the performance of this platform to conventional static 3D drug testing systems (standard 384-well plates) and evaluated its predictive potential at a small scale. Additionally, using single-cell RNA sequencing (scRNA-seq), we investigated drug response mechanisms across static and microfluidic conditions. This approach aims to improve the predictive accuracy of preclinical testing and support the development of more effective, individualized cancer treatments.

RESULTS

Recapitulating endothelial barrier physiology in an open-top TOC

To study the efficacy of individual cancer drugs in tissue contexts, such as those treated with intravenously (IV) administered chemotherapeutics, we used an open-top microfluidic platform featuring a plate-format chip design (AKITA). This system allows for semi-high throughput analysis of multiple samples simultaneously, and facilitates easy cell recovery for downstream assays, such as scRNA-seq (Figure 1A). Since the formation of flow is based on automated rocking, the system does not require complicated or expensive equipment. The two compartments in the chip are connected by a porous membrane, allowing selective exchange between them and enabling rapid, cost-effective analysis of various sample types or multiple drug treatments.

In this study, we developed an approach using the chip system to monitor tumor responses to treatments delivered through microchannels in the absence or presence of a built-in endothelial barrier. Controlled slow rocking was employed to simulate shear stresses induced by liquid flow, a condition necessary for the formation of a proper endothelial monolayer. According to previous studies, average human physiological vascular stresses, ranged from 2 to 7 dyn/cm^2, regulate diverse cellular responses and their flow-dependent behaviors, including protein expression and cell morphology.[13,14] In our experimental settings, the shear stress

CellPress
OPEN ACCESS

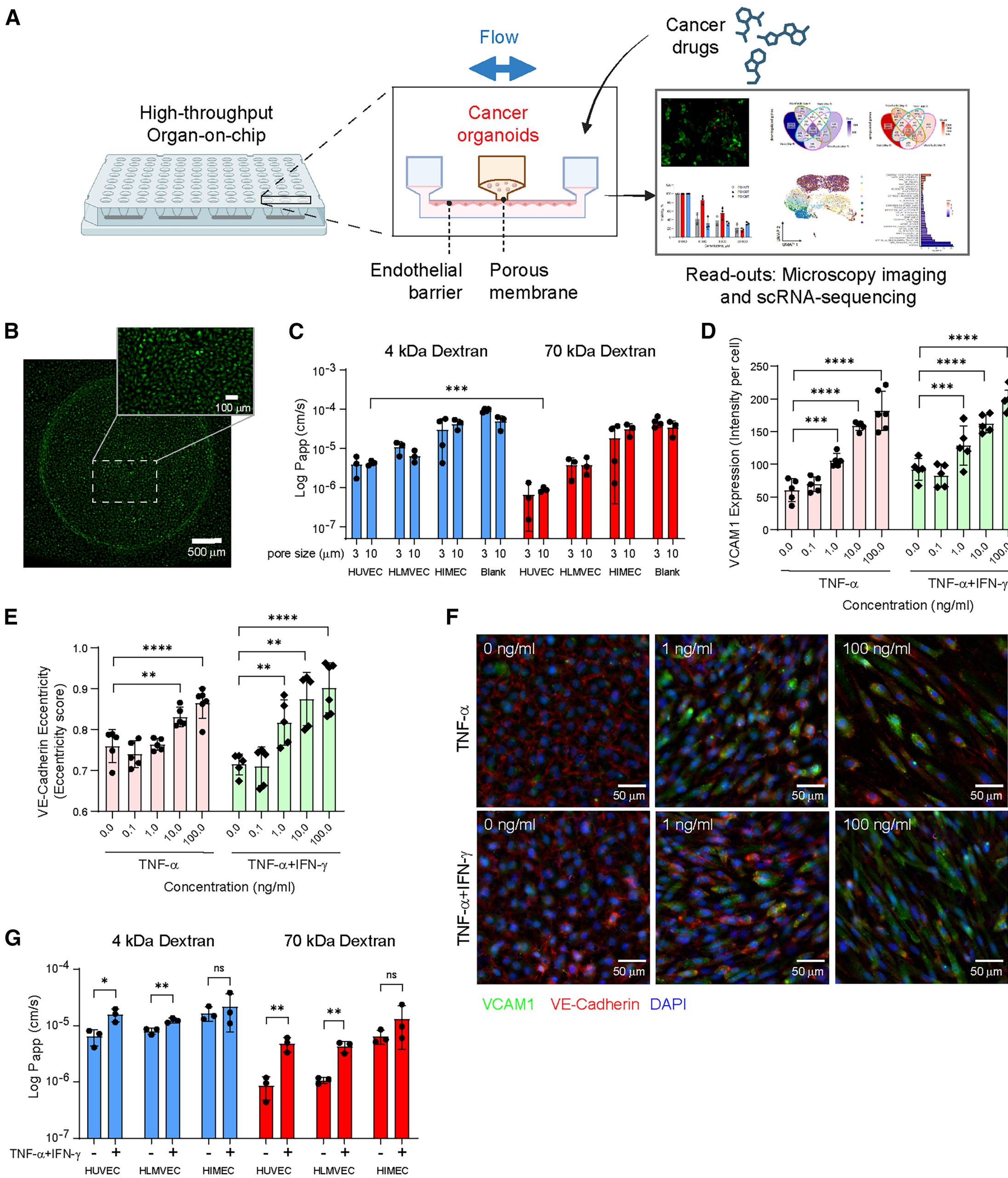

Figure 1. Evaluation of various endothelial barriers built inside the microfluidic TOC

(A) Schematic of the AKITA microfluidic platform, with open-top chambers containing patient-derived organoids in Matrigel as tumor sites and microchannels coated with endothelial cells simulating vessels. Drugs can be added to the reservoirs, and cell recovery is simplified by the open-top design. Fluorescence microscopy and scRNA-seq were used for analysis. Image was created using BioRender. (B) HUVEC endothelial barrier stained with calcein-AM viability dye. Magnification 10×, scale bars: 500 (main) and 100 μm (zoom).

(C) Effect of membrane pore size (3 vs. 10 μm) on HUVEC, HLMVEC, and HIMEC monolayer formation and permeability to 4 and 70 kDa dextran. Apparent permeability (dextran accumulation in open-top chamber) was measured after 120 min.

(D–F) Effect of cytokines (TNF-α or TNF-α+IFN-γ) on HUVEC morphology and inflammation, assessed by VCAM1 expression (D), cell eccentricity (E), and fluorescence imaging (F). Cytokine concentrations: TNF-α (50 ng/mL) and TNF-α+IFN-γ (50 ng/mL each) for 48 h. Statistical analysis: one-way ANOVA (***p*: 0.0017–0.0072; ****p*: 0.0001–0.0006; and *****p* < 0.0001). Scale bars, 50 μm.

(G) Inflammation effects on barrier permeability, assessed using dextran after 24 h incubation with TNF-α+IFN-γ. Sample size: 3 per condition, data shown as mean ± SD, unpaired two-tailed *t* test (**p*: 0.0255; ***p*: 0.0044; ***p*: 0.0090; and ***p*: 0.0092). "Blank" is a no-cell control condition (C).

See also Figure S1.

that exerts on endothelial monolayer is about 2.5 dyn/cm^2 (0.25 Pa) (Figure S1A).

Furthermore, it was shown that undisturbed liquid flow promotes cell resilience and supports the formation of proper cell-cell junctions.[15,16] Based on these findings, we hypothesized that drug administration through the microchannel could replicate the route chemotherapy drugs take when entering tissue via the vasculature. The structure and properties of the endothelial barriers vary between different organs. For example, brain capillaries form tighter junctions than microvessels in the liver, bone marrow, or lungs. Under normal conditions, most microvessels are impermeable, but this permeability is altered in pathological states, such as inflammation and cancer, where endothelial signaling cascades increase vascular permeability and facilitate leukocyte extravasation.[16]

To understand how the source of endothelial cells impacts barrier permeability, we compared the barrier integrity formed by human umbilical vein endothelial cells (HUVECs), human lung microvascular endothelial cells (HLMVECs), and human intestinal microvascular endothelial cells (HIMECs). In general, the endothelial cells formed complete monolayers inside the microfluidic channel approximately after 4–5 days of culture under slow rocking conditions (Figure 1B). Time required for monolayer to reach full confluency was assessed during initial optimization studies using the transepithelial/transendothelial electrical resistance (TEER) adapter and used as a reference in the following experiments.[17] Barrier permeability was assessed by injecting high- or low-molecular weight fluorescent dextran into the microfluidic channel and measuring their penetration into the top chamber through the artificial vasculature. During initial tests, we measured dextran diffusion through the bare membrane and selected the appropriate incubation time for the following experiments based on these results (Figure S1B). We observed that each endothelial cell source formed a monolayer with distinct permeability, with minimal impact from the membrane pore size (Figure 1C). HUVECs formed the tightest barrier, consistent with their origin from large placental vessels, where strong junctions maintain vascular integrity. HIMECs were the leakiest, reflecting the naturally higher permeability of intestinal microvessels required for nutrient absorption and immune cell trafficking. Smaller dextran penetrated the HUVEC barrier slightly better than larger ones, though the difference was not substantial.

Inflammation, induced by tumor necrosis factor-alpha (TNF-α) or TNF-α combined with interferon-gamma (IFN-γ), led to endothelial cell activation, as shown by upregulation of vascular cell adhesion molecule-1 (VCAM1), a key adhesion molecule associated with inflammatory signaling (Figures 1D–1F and S1C). This was accompanied by changes in cell morphology, including increased elongation and disrupted cell-cell contacts. Morphological alterations, quantified by increased cell eccentricity (a measure of deviation from circularity), were more pronounced in cells treated with the TNF-α+IFN-γ mixture. As expected, inflammation also increased barrier permeability, with the most pronounced effects observed in HUVEC and HLMVEC cells in a concentration-dependent manner (Figures 1G, S1D, and S1E). These findings align with known endothelial responses to inflammatory cues, including weakened junction integrity and increased paracellular transport.

Our results demonstrate that the TOC microfluidic system can recapitulate key physiological features of endothelial barriers from different vascular beds, allowing for *ex vivo* investigation of endothelial permeability and responses to drugs or inflammation. For the following experiments, we decided to use HUVECs since they are the most widely used cells in OOC modeling and also formed the most robust and reproducible endothelial barriers in the TOC. This choice of endothelial cells source facilitates comparison of our results with prior work in the field.

Drug sensitivity is modulated by the administration route in a subset of models

Building on our characterization of endothelial barrier physiology and permeability, we next used the model to study the ingress of chemotherapeutic drugs into the tumor site via vascular extravasation. In addition to establishing an endothelial barrier, patient-derived tumor organoids from lung and pancreatic cancers were embedded in extracellular matrix in the top chamber to simulate the tumor microenvironment.

To evaluate the platform's ability to model clinically relevant drug exposure routes, such as intravenous (IV) administration or depot tablets, we compared chemotherapy responses under different culture conditions and administration methods to assess how well the system mimics drug delivery through the bloodstream. Organoids were cultured either in a standard 384-well plate (traditional setup for drug testing experiments) or in the TOC system, and chemotherapeutics were delivered either statically on top of the organoids in the 384-well plate ("static") or via the microchannel, in the absence or presence of an endothelial barrier ("microfluidic" or "intravenous," respectively), simulating vascular drug delivery route (Figure 2A). HUVEC cells were used to create endothelium, as they formed the most robust barriers with pronounced responses to cytokine-induced inflammation.

Both lung and pancreatic cancer organoids were tested with their respective standard-of-care chemotherapy regimens. When cell viability was measured after treatment, we observed that in specific models (ORG55 and PO126T) the organoids were more resistant to chemotherapy when drugs were administered through the microchannel under flow conditions, compared to static administration on top of the organoids, which mimics traditional drug testing scenarios (Figures 2B–2E). In these specific models, this resistance was even more pronounced when drugs had to penetrate the endothelial barrier to reach the tumor site.

Noticeably, the viability of endothelial cells and permeability of cell monolayer were not affected by administered chemotherapeutics (Figures S1F and S1G). Furthermore, the TOC system highlighted apparent inter-patient variability in chemotherapy efficacy (Figures 2B–2E and S2A). These results highlight the importance of drug administration route and culture conditions in determining therapeutic efficacy, at least in some models.

Cytokine-mediated infiltration of immune cells into the tumor site

Given the significance of immunotherapies in modern cancer treatment, we next aimed to evaluate the applicability of the TOC for studying immune cell migration and efficacy using

CellPress
OPEN ACCESS

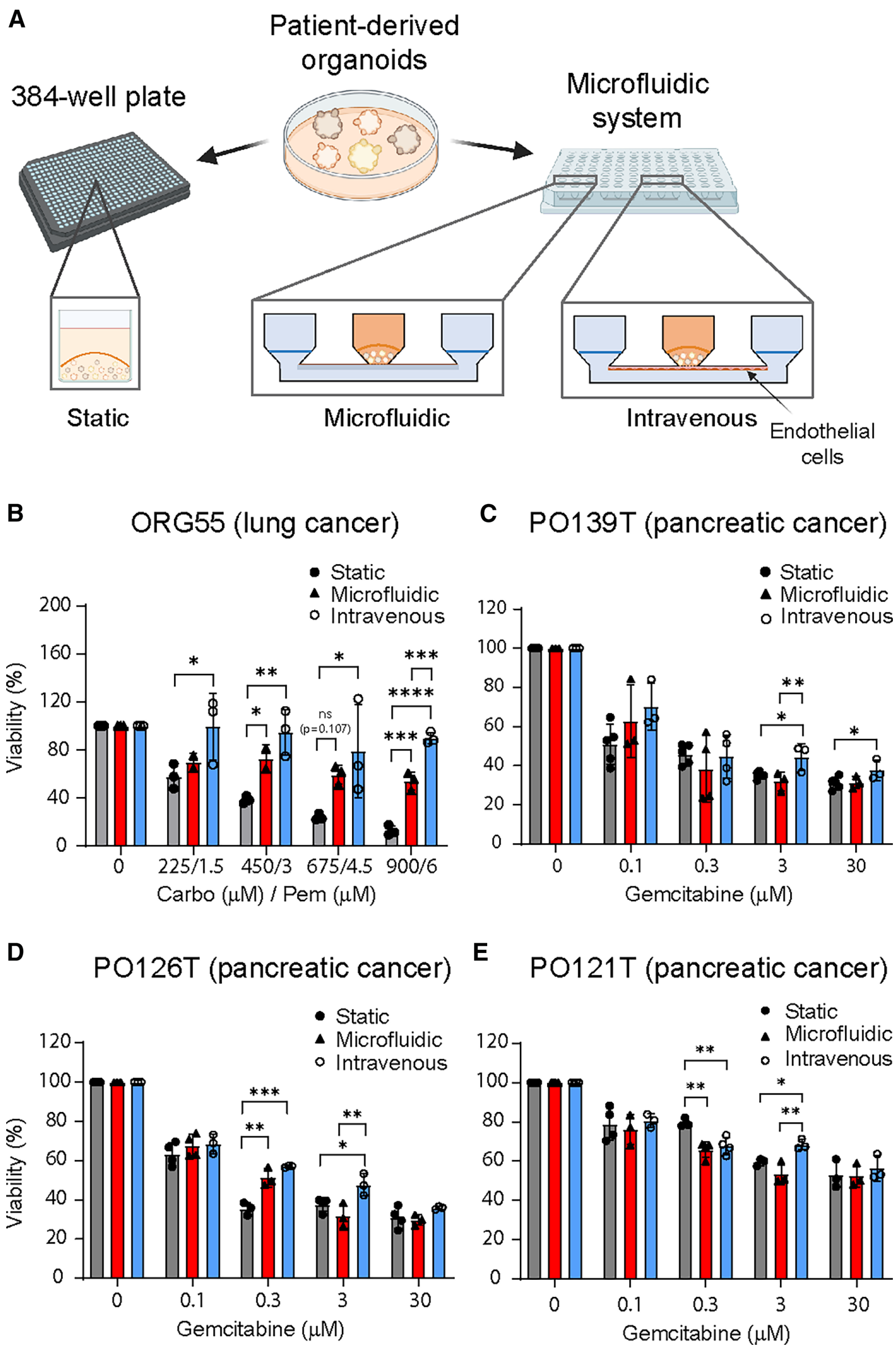

Figure 2. Applicability of TOC to drug response assessment

(A) Schematic of drug administration in cancer organoid models: "static" (384-well plate), "microfluidic" (open-top chambers and bare micro-channel), and "intravenous" (open-top chambers and endothelial barrier).

(B–E) Cell viability of lung ORG55 (B) and pancreatic PO139T (C), PO126T (D) and PO121T (E) organoids after 3-day treatments with carboplatin-pemetrexed (lung) or gemcitabine (pancreatic) at varying concentrations. Data shown as mean ± SD (three replicas), one-way ANOVA (ORG55: *p: 0.044, *p: 0.0366, **p: 0.0034, ns: 0.107, *p: 0.0265, ***p: 0.0001–0.0002, and ****p: < 0.0001; PO139T: *p: 0.0182–0.0408 and **p: 0.0075; PO126T: *p: 0.0328, **p: 0.0012–0.0056, and ***p: 0.0002; and PO121T: *p: 0.0252 and **p: 0.0016–0.004) was used to assess statistical significance.

See also Figure S2.

oncolytic adenoviruses, promising tools engineered to selectively destroy cancer tissue and restore antitumor immune response in cancer patients via cytokine release.[18–20] To investigate this, we used pancreatic cancer organoids (PO126T) embedded in an extracellular matrix and seeded into the open-top chambers of the TOC, in the absence of an endothelial barrier. These organoids were exposed to either a non-armed oncolytic adenovirus or a C-X-C motif chemokine ligand 10 (CXCL10)-expressing virus to increase cytokine-based immune cell attraction.[21] Labeled peripheral blood mononuclear cells (PBMCs) were then introduced into the microfluidic channels, and their migration toward the cancer organoids was assessed using fluorescence microscopy (Figures 3A and 3B).

Both the non-armed and CXCL10-expressing viruses increased immune cell accumulation within the organoid-containing matrix, whereas non-infected organoids showed only minor immune cell recruitment (Figures 3A, 3C, and S2B–S2D). These results suggest that baseline cytokine levels produced by the cancer cells were limited in their ability to promote immune cell migration. Notably, viral transfection led to visible oncolysis around the 72-h time point, which likely contributed to the increased immune infiltration observed (Figure 3A). Given that the pancreas is known to have a more immunosuppressive microenvironment than many other tissues, we compared the immune response in the pancreatic cancer model with other tumor types with potentially more immunogenicity. To establish this, we tested two additional cancer models (lung cancer; ORG96T and mesothelioma; Meso15) that may elicit a more robust immune reaction (Figures 3B, 3D, and 3E).

Among the three models, the lung cancer model attracted the highest number of immune cells on its own, even before transfection with the non-armed oncolytic adenovirus, while the other two models showed minimal baseline immune cell migration. This may reflect the inherently higher immunogenicity of lung tumors compared to pancreatic and mesothelioma tumors. Immune cell migration induced by the inflammation from the non-armed virus at 48 h, was highest in the mesothelioma model and lowest in the pancreatic cancer model, while the

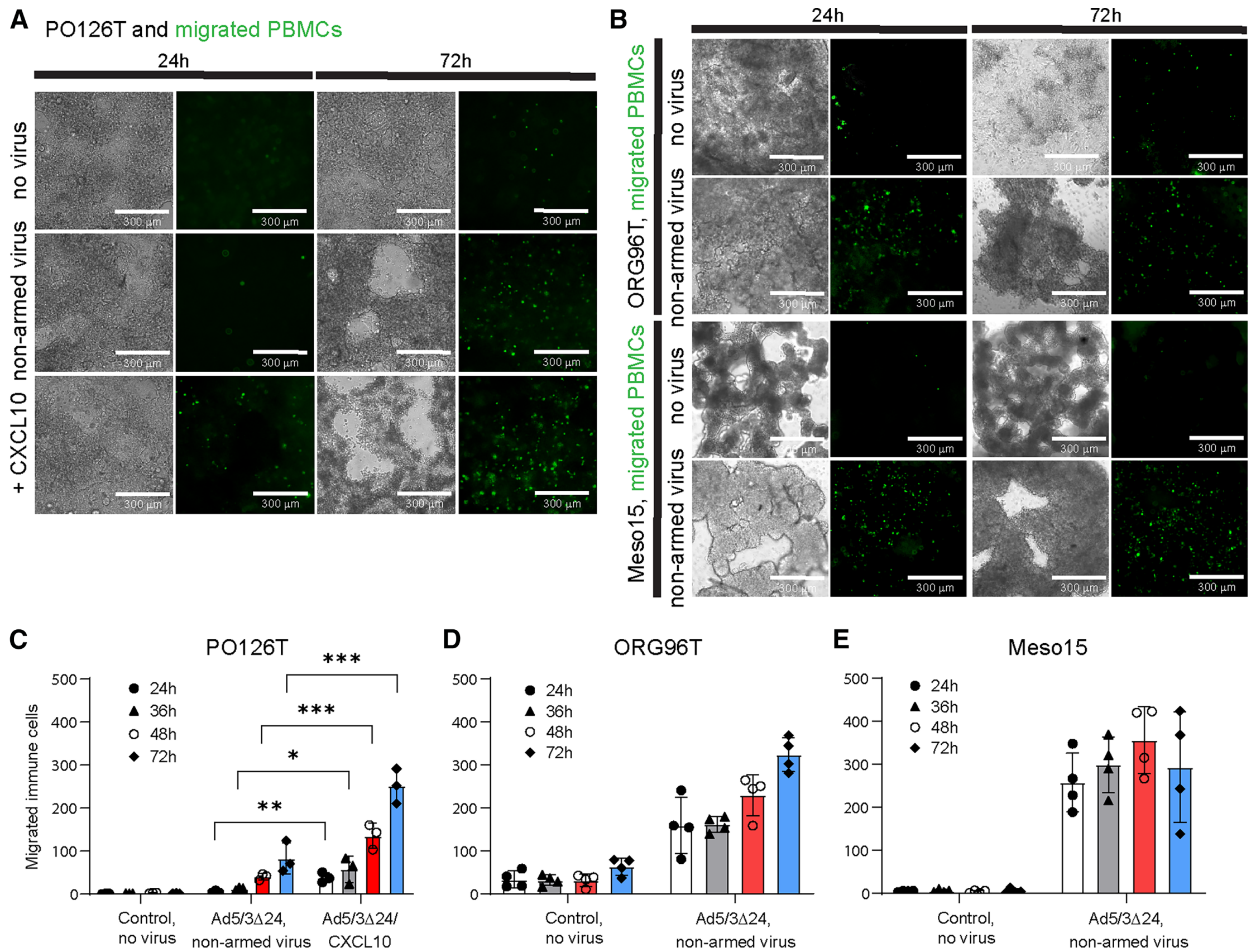

Figure 3. Applicability of TOC to immune cell infiltration studies

(A and B) BF and fluorescence images of organoids and labeled PBMCs at 24 and 72 h post-infection with Ad5/3Δ24 (non-armed) or Ad5/3Δ24/CXCL10 (CXCL10-expressing) viruses for PO126T (A), ORG96T, and Meso15 (B). Labeled PBMCs were added to microchannels and imaged at 24, 36, 48, and 72 h. Scale bars, 300 μm.

(C–E) Quantification of PBMC migration to PO126T (C), ORG96T (D), and Meso15 (E) organoids post-transfection with Ad5/3Δ24 or Ad5/3Δ24/CXCL10 viruses. Data shown as mean ± SD (3–4 replicas), one-way ANOVA (*p: 0.0219, **p: 0.001, and *p: 0.0005–0.0006). Cell counts at 48 h for ORG96T without transfection (30 ± 14), and in the presence of non-armed virus transfection for ORG96T (229 ± 48), Meso15 (356 ± 77), and PO126T (40 ± 7).

See also Figure S2.

lung cancer model showed a moderate increase in cell migration (Figures 3C–3E). These differences might be explained by distinct tumor characteristics, such as variations in cytokine secretion, that influence immune cell recruitment. Our results suggest that the TOC system can model organ-specific immune cell migration toward cancer organoids and that this process can be enhanced by virus-induced inflammation.

Co-clinical modeling of chemotherapy responses suggests predictive potential

Although the clinical predictivity of tumor organoids has been explored in previous studies,[22–25] the clinical relevance of TOC models remains largely unexplored. To address this, we compared the predictive capacity of the TOC platform with that of static organoid cultures by performing retrospective co-clinical drug testing using pancreatic cancer organoids alongside corresponding patient follow-up data (Table S1).

We assessed organoid treatment responses under both static and microfluidic conditions. Chemotherapy concentration ranges were selected based on a calculated match between clinically relevant patient doses used during treatment in the clinic (Table S1) and those used *in vitro.*[26] The results were consistent with our previous observations, indicating a generally higher level of primary drug resistance in the TOC (Figure 4).

Both systems were able to register drug dependent decrease in viable cells induced by chemotherapeutics, but the response in TOC for a subset of models (e.g., PO126 and PO121T), was more linear (less binary) potentially due to the necessity for the drug to diffuse from the microchannel into the chamber containing cancer organoids (Figures 4A and 4B). This effect was even further strengthened by the presence of the endothelial barrier (Figure 4C). Based on our observations, we hypothesized that the drug administration through the microchannel could be more physiologically relevant and comparable with drug distribution in patient tissue, where it is delivered through blood stream and must diffuse to the cancer site through the vessel wall.

The testing results demonstrated a correlation with clinical outcomes (Table S1). Models PO109T and PO121T exhibited the highest cell viability at clinically relevant doses of gemcitabine (∼3 μM), indicating poorer drug response. In contrast,

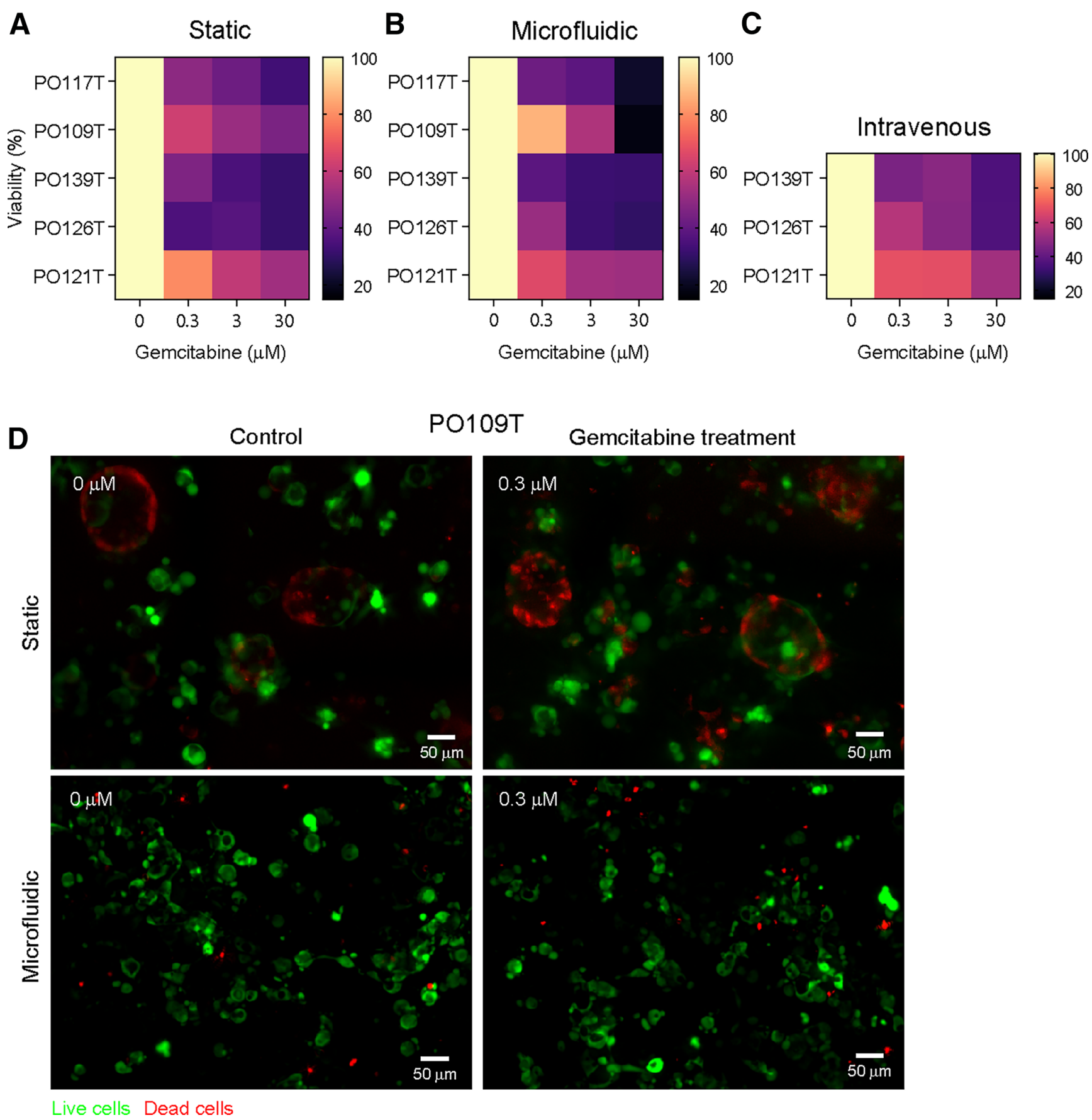

Figure 4. Interpatient comparisons of responses to drugs in standard vs. microfluidic conditions

(A–C) Five (A and B) and three (C) types of pancreatic cancer organoids treated with gemcitabine for 3 days in 384-well plate ("static") or TOC system without and with endothelial barrier ("microfluidic" and "intravenous", respectively). Cell viability (live/dead) was analyzed at varying drug concentrations and presented using heat maps where 100 means percentage of viable cells in the absence of a drug. Sample sizes: 3 in microfluidic, 4–5 in static.

(D) Microscopy images of PO109T in "static" and "microfluidic" conditions with (0.3 μM) or without (0 μM) gemcitabine. Live cells (green, calcein-AM) and dead cells (red, propidium iodide) are shown. Scale bars, 50 μm.

PO117T, PO139T, and PO126T demonstrated greater *ex vivo* sensitivity to gemcitabine.

Consistent with these observations, patient PO109T experienced suspected tumor recurrence and subsequently passed away (overall survival [OS]: 640 days). Treatment of patient PO121T was discontinued after thorough clinical assessment concluded that no benefit would be expected from further therapy, despite dose escalation, due to poor response (OS: 253 days). Patient PO117T demonstrated longer OS (1,205 days) and was cancer-free at follow-up assessment 1,031 days from treatment initiation (as of October 10, 2024); however, treatment was later discontinued due to elevated liver enzyme levels, and the patient subsequently passed away.

Patient PO139T remains alive (1,296 days from treatment initiation, as of November 11, 2025) with stable disease during treatment cycles, although signs of disease progression were observed during three treatment breaks. For patient PO126T, *ex vivo* TOC results demonstrated gemcitabine efficacy at clinically relevant doses (∼3 μM), which correlated with partial response observed during second-line therapy. However, treatment was discontinued due to drug-related pulmonary toxicity, after which disease progression occurred (OS: 637 days).

In PO126 model, the drug response in static culture saturated already at relatively low concentrations, making it difficult to resolve potential dose-dependent differences. In contrast, the intravenous TOC format showed a more gradual response across the tested concentrations, consistent with drug exposure being limited by transport and barrier effects. Interestingly, the clinically administered gemcitabine dose for this patient was increased during treatment, illustrating how delivery-constrained responses may be relevant when considering dose-response relationships under physiologically limited drug exposure.

Based on the TOC results, several potential advantages of this system can be highlighted. First, TOC can mimic slow drug penetration into tumor tissue, resembling *in vivo* conditions. Second, it enables assessment of the necessity and potential benefit of dose escalation or reduction. Given that treatment-related toxicity remains a major limitation of chemotherapeutic regimens, TOC may serve as a useful tool to support patient-centric optimization of treatment dosing strategies. Nevertheless, further validation in a larger patient cohort is required.

Cell signaling is profoundly impacted by the culture system

Unlike many other OOC platforms, the open-top design of the TOC enabled recovery of surviving cells after chemotherapy treatment for gene expression analysis by pooled scRNA-seq. We used lung (ORG2), and pancreatic cancer (PO109T) models treated with either vehicle or standard-of-care chemotherapy for 4 or 7 days under static (in 384-well plate) or microfluidic conditions (in TOC without endothelial barrier). Uniform manifold approximation and projection (UMAP) analysis revealed substantial differences between static and TOC cultures in both control and chemotherapy-treated groups (Figures 5A and 5B). Further pathway analysis of untreated samples revealed distinct differences between fluidic and static cultures. In TOC, metabolic pathways such as oxidative phosphorylation (OXPHOS) and fatty acid metabolism were upregulated, while glycolysis and hypoxia-related pathways were downregulated (Figures 5C and 5D). These

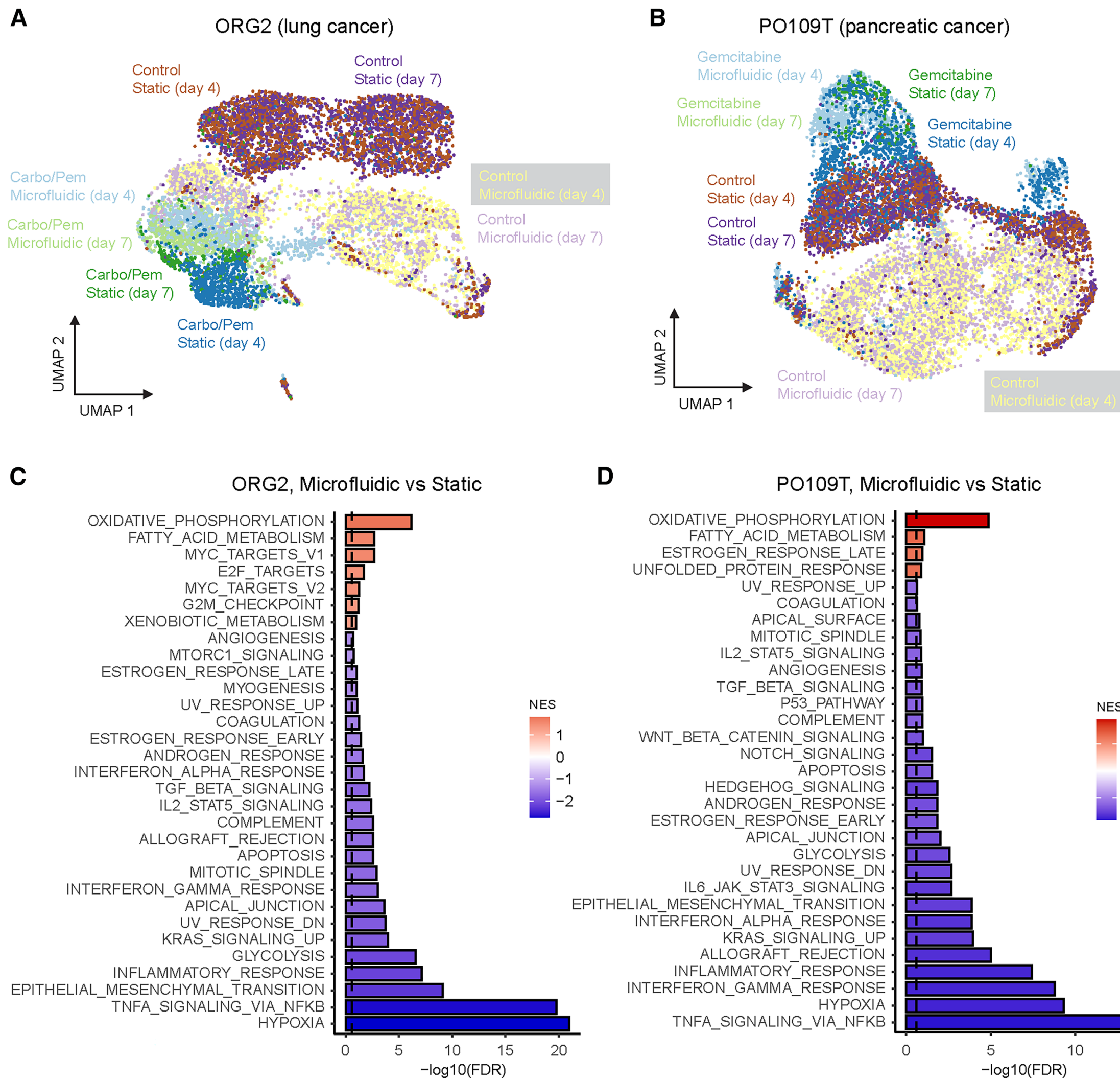

Figure 5. Culturing conditions induce transcriptional changes in tumor cells

Culturing of organoids in TOC significantly changes transcriptional activity of multiple genes. Pooled scRNA-seq was used to compare transcriptional profiles of two organoid models cultured in microfluidic or static conditions.

(A and B) UMAP plots for ORG2 (A) and PO109T (B) show distinct cell clustering for each culturing conditions both in the presence and absence of chemotherapy.

(C and D) Pathway analysis of days 4 and 7 combined control samples (without drug treatment) between microfluidic and static conditions revealed significant switch in multiple metabolic pathways for both models, ORG2 (C) and PO109T (D). Red-colored bars indicate upregulation of corresponding pathways, whereas blue is downregulation.

results are in line with previous observations described in microfluidic and OOC studies under normoxic conditions.[27]

Additionally, key signaling pathways including TNF-α signaling via nuclear factor kappa-light-chain-enhancer of activated B cells (NF-kB) and other inflammatory responses were reduced in TOC compared to static conditions, likely reflecting improved nutrient and oxygen availability and reduced cellular stress in the microfluidic environment. Based on these results, we suggest that the TOC system can capture physiologically relevant metabolic processes and enable analysis of pathway alterations in a simple way by using scRNA-seq.

Oncogenic pathways are known to promote OXPHOS to support cancer cell metabolism and survival, which can lead to increased reactive oxygen species (ROS) production.[28] Drug-resistant cancer cells often exhibit high OXPHOS activity but rely on antioxidant defense mechanisms regulated by nuclear factor erythroid 2-related factor 2 (NRF2) to detoxify ROS and enhance survival.[28] Given the observed metabolic differences, we next examined how culture conditions influence drug response-related signaling following chemotherapy treatment. The aim of this comparison was to assess if alterations in cell responses due to culture conditions can be more apparent in TOC system and if the TOC could be potentially used as a preliminary screening system for resistance development.

Many differentially expressed genes were shared between static and fluidic systems, while others were also uniquely

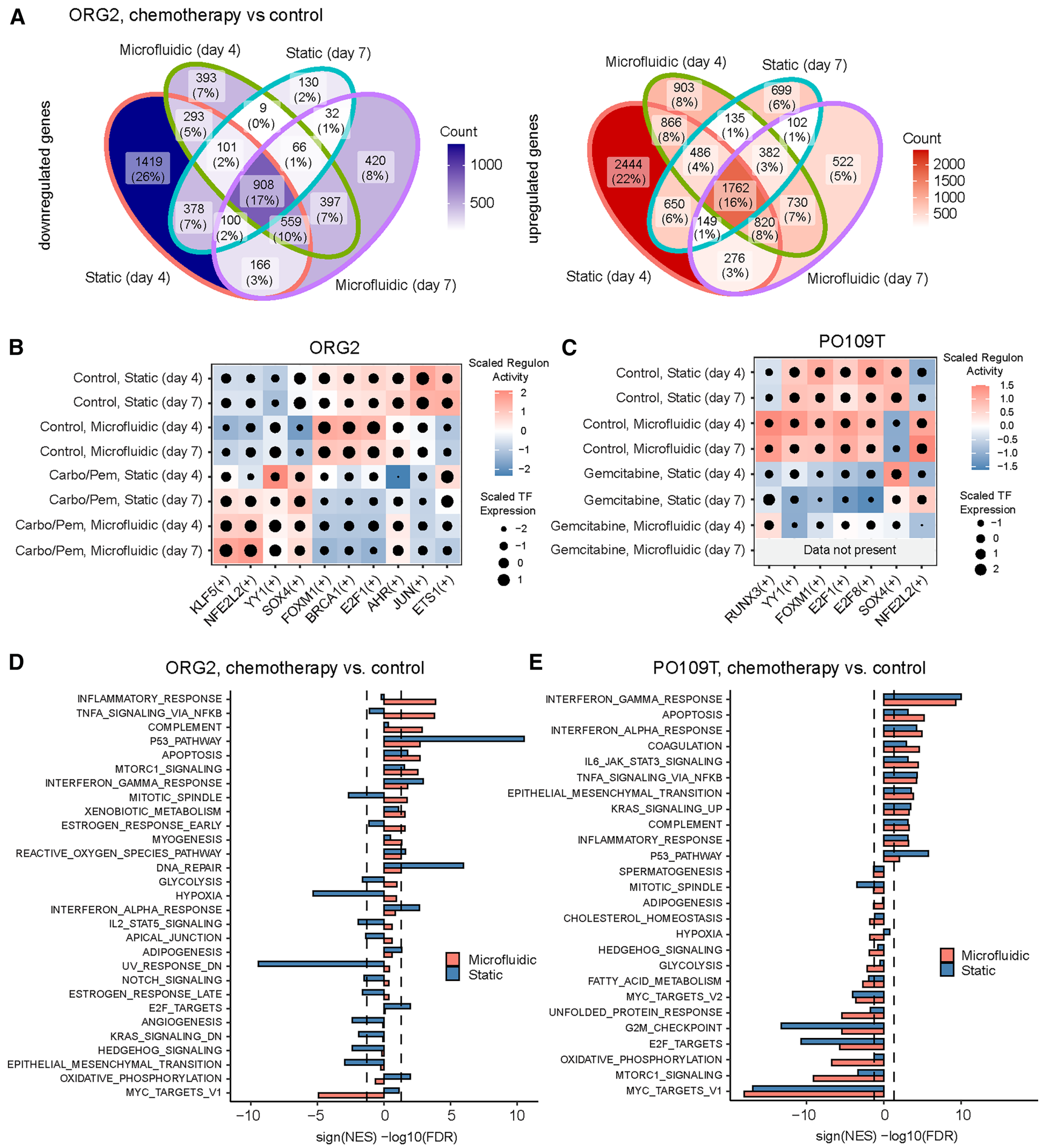

Figure 6. Chemotherapies have different effects on gene expression of tumor cells depending on culturing conditions

(A) Schematic representation of total gene numbers (common and unique) that were downregulated (blue scale) or upregulated (red scale) in microfluidic vs. static conditions in the absence or presence of drug treatment for ORG2 model.

(B and C) Comparison of selected transcriptional factors (TF) expression and regulon activity scores for ORG2 (B) and PO109T (C) depending on culturing conditions and drug treatment. Color coding represents increased (red) or decreased (blue) scaled regulon activity, whereas size of black circles correlates with scaled TF expression intensity.

(D and E) Effect of four-day chemotherapy treatment on the pathway activity for ORG2 (D) and PO109T (E) depending on cultured conditions (microfluidic or static). Color coding of the bars: pink, culturing in microfluidic conditions; and light blue; in static conditions. Bars directed to the right (+) show upregulating activity of genes in corresponding pathways, and to the left (–) show downregulation in their activity. Some of the pathways demonstrate opposite responses to chemotherapy treatment received in static or microfluidic setups.

See also Figure S3.

altered in a single condition (Figures 6A and S3A). Notably, transcription factor regulon analysis revealed increased NRF2 (*NFE2L2*) activity in ORG2 within the fluidic system following chemotherapy treatment, suggesting an enhancement of antioxidant defense response (Figures 6B. 6C, S3, S3B, and S3C). In the pancreatic cancer model, although *NFE2L2* was upregulated in the fluidic control condition compared to static, it was not induced by the treatment, suggesting alternative

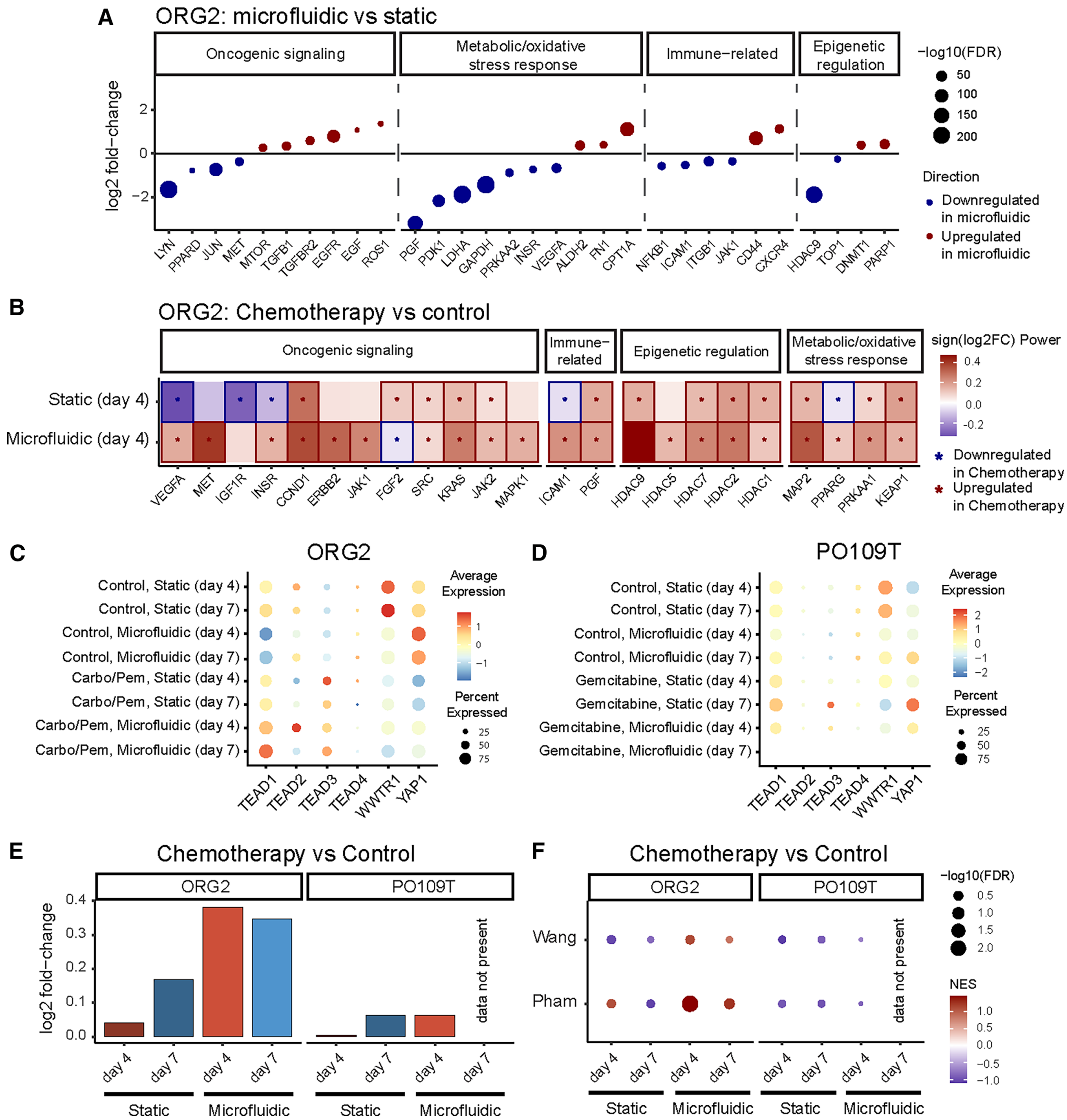

Figure 7. Pharmacologically targetable genes under static vs. microfluidic conditions and chemotherapy

(A) Genes in oncogenic, metabolic/oxidative stress response, immune-related, and epigenetic regulation pathways show differential expression between microfluidic and static conditions for ORG2. Red and blue dots represent upregulated and downregulated genes in microfluidic conditions, respectively, with spot size indicating FDR.

(B) Chemotherapeutic treatments cause different gene expression changes in static vs. microfluidic conditions for ORG2, with significant differences highlighted in square frames.

(C and D) Expression of TEAD family transcription factors in ORG2 (C) and PO109T (D) models under static/microfluidic conditions with/without chemotherapy. Color indicates upregulation (red) or downregulation (blue), and spot size correlates with gene expression prevalence.

(E) TEAD1+ regulon activity scores in response to chemotherapy for ORG2 and PO109T after 4 (red) or 7 (blue) days of treatment in static/microfluidic settings.

(F) GSEA of TEAD signatures in response to treatments, with upregulation (red) and downregulation (blue) shown as normalized enrichment score (NES) and spot size indicating FDR.

See also Figure S3.

pathways for OXPHOS tolerance in this model. In ORG2, chemotherapy treatment also led to increased Krüppel-like factor 5 (KLF5) activity, more prominently in the TOC (Figure 6B). KLF5 has previously been linked to chemotherapy resistance by promoting cell survival.[29]

Pathway responses also varied in the two systems upon treatment, particularly in the lung cancer model, where chemotherapy differentially affected TNF-α signaling, mitotic spindle signaling, glycolysis, hypoxia, OXPHOS, and MYC signaling (Figures 6D and 6E). Interestingly, p53 and DNA damage

signaling were more activated under static conditions upon treatment in ORG2, with similar patterns observed for p53 signaling in PO109T.

Together, these results demonstrate that the TOC system modulates transcriptional programs related to metabolism and drug response, including factors such as NRF2 and KLF5, which may influence chemotherapy tolerance.

Targetable gene expression patterns differ under static and fluidic conditions

Finally, to understand how culture conditions could impact drug testing outcomes, we analyzed how the culture environment specifically impacts the expression of pharmacologically targetable genes identified in the DrugBank database. In untreated conditions, we observed significant differences in targetable gene expression between microfluidic and static conditions (Figures 7A and S3D). We categorized the most relevant targetable genes into four groups: oncogenic signaling, metabolic/oxidative stress response, immune-related, and epigenetic regulation. Interestingly, some expression changes were shared between the two models, such as higher expression of placental growth factor (*PGF*; angiogenesis), insulin receptor (*INSR*; metabolic and proliferative signaling), intercellular adhesion molecule 1 (*ICAM1*; immune cell trafficking and inflammation), and histone deacetylase 9 (*HDAC9;* epigenetic regulation) in static cultures. However, other expression changes were unique to each tumor model, suggesting either organotypic or patient-specific variability in response to flow. For instance, epidermal growth factor (*EGFR*; oncogenic signaling) and fibronectin 1 (*FN1*; extracellular matrix remodeling) were downregulated in PO109T but upregulated in ORG2 (Figures 7A and S3D).

The models also exhibited distinct targetable gene expression responses to chemotherapy (Figures 7B and S3E). In ORG2, genes such as vascular endothelial growth factor (*VEGFA*; angiogenesis), mesenchymal epithelial transition factor proto-oncogene receptor tyrosine kinase (*MET*; cell growth and motility), insulin-like growth factor receptor (*IGF1R*; cell proliferation and survival), insulin receptor (*INSR*), intercellular adhesion molecule 1 (*ICAM1*), and peroxisome proliferator-activated receptor gamma (*PPARG*; immune regulation and metabolism) were downregulated in static conditions but upregulated in fluidic system (Figure 7B). Conversely, fibroblast growth factor 2 (*FGF2*; cell growth and angiogenesis) showed an opposite pattern.

Overall, while many genes of interest were induced by chemotherapy in both conditions, the magnitude of induction was greater in the fluidic cultures. These results highlight that culture conditions can substantially influence gene expression patterns relevant for drug testing, with potential implications for evaluating single and combination therapies. In other words, patient-specific responses to chemotherapy can be more apparent in TOC that could lead to more personalized treatment selection.

Culture conditions influence YAP1-mediated drug resistance signaling

Yes-associated protein 1 (YAP1), a key component of the Hippo signaling pathway, plays a crucial role in drug resistance across various cancer types and treatments.[30] YAP1 manifests its effects through interaction with the transcriptional enhanced associate domain (TEAD) family of transcription factors (TEAD1-4) and the transcriptional co-activator with PDZ-binding motif (TAZ, *WWTR1*), which together regulate downstream processes such as cell growth, proliferation, migration and survival. Given the higher chemotherapy resistance observed in the fluidic culture system, we next analyzed the expression patterns of YAP1, TAZ, and TEAD family members in cancer organoids cultured under static and fluidic conditions. It is known that YAP1 is sensitive to matrix stiffness, cell density, mechanical stretching, and frictional force characteristic of flow in lymphatics.[31] Upregulation of its activity in response to shear stress is ingrain attribute of various cancers and correlates with increased cell migration and adverse prognosis.[31,32]

Our experiments demonstrated significant difference in gene expression prior to drug treatment, particularly in the lung cancer organoid model (ORG2). Specifically, *TEAD1* and *WWTR1* were downregulated under fluidic conditions, while *YAP1* was upregulated (Figure 7C). Following chemotherapy treatment, ORG2 exhibited increased expression of TEAD1 and TEAD3, alongside downregulation of WWTR1 and YAP1 transcripts. Notably, *TEAD1* expression was more upregulated by chemotherapy in the fluidic system compared to static conditions. This pattern suggests that YAP1 signaling is initially more active in the fluidic system, which is consistent with prior reports, supporting the benefits of TOC compared to traditional static platforms. Although *YAP1* transcript levels decrease after treatment, *TEAD1* expression increases, possibly reflecting compensatory regulation or post-translational mechanisms that maintain TEAD-driven transcription and drug resistance signaling. However, as these data are based on transcript levels, it remains critical to investigate corresponding protein expression, localization, and activity to fully understand the functional implications.

In contrast, the pancreatic cancer model (PO109T) showed only mild differences in *WWTR1* and possibly *YAP1* expression between culture conditions, and chemotherapy had minimal impact on TEAD family member expression (Figure 7D). These observations suggest that the lung cancer model may rely more heavily on the YAP/TEAD transcriptional axis for primary drug resistance, whereas the pancreatic model likely employs alternative resistance mechanisms that warrant further investigation.

To assess whether expression changes translated into altered transcriptional activity, we examined the regulon activity of TEAD1. Chemotherapy treatment led to a significant increase in TEAD1 regulon activity in ORG2, particularly within the TOC. In contrast, PO109T showed only a mild increase in regulon activity post-treatment (Figure 7E). Since YAP signaling is mediated by a complex interplay between YAP, TAZ, and TEAD transcription factors, we further performed gene set enrichment analysis (GSEA) using two established TEAD activity signatures (Wang signature; 22 genes and Pham signature; 139 genes, Table S2) to compare chemotherapy responses under static versus fluidic conditions (Figures 7E and 7F). GSEA revealed significant upregulation of TEAD-associated gene sets after chemotherapy exclusively in the fluidic system of ORG2, but not in PO109T, reinforcing the notion of distinct drug response pathways between these models (Figure 7F).

Taken together, these findings demonstrate that culture conditions profoundly influence YAP/TAZ-TEAD signaling and associated drug resistance mechanisms. Our data also highlight the importance of incorporating physiologically relevant culture systems, like fluidic culture, when modeling treatment outcomes, to better capture tumor-specific resistance pathways.

DISCUSSION

Our study demonstrates the utility of a precision TOC model for investigating the complex interactions between tumors and the vascular environment, and for evaluating the efficacy of drug permeability and immune cell delivery. The model's open-top microfluidic design enables multiplexed sample analysis, straightforward cell retrieval for downstream assays, and compatibility with automation. Unlike traditional cultures, the platform incorporates an endothelial barrier mimicking the vascular wall, enhancing physiological relevance for drug delivery studies. Controlled rocking simulates blood flow, supporting robust endothelial monolayer formation and allowing organ-specific vascular phenotypes to be replicated. Endothelial cells from diverse origins generated barriers with distinct permeabilities, reflecting physiological variability observed *in vivo* and underscoring the importance of barrier source selection. Furthermore, the TOC recapitulates pathological states such as inflammation through cytokine-induced modulation of endothelial permeability. Collectively, these features highlight the TOC's capacity to emulate dynamic tumor-vascular crosstalk.

Immune cell infiltration into the tumor microenvironment is strongly associated with immunotherapy response rates and patient prognosis.[33–35] In our pancreatic cancer model, limited immune cell migration in the TOC aligned with the immunologically "cold" nature of pancreatic tumors, characterized by a low mutational burden and dense extracellular matrix.[33] In contrast, malignant pleural mesothelioma is associated with a dynamic immune microenvironment exhibiting both immuno-suppressive and immuno-activating properties,[34] which could explain the results observed with the mesothelioma model during inflammation induced by virus transfection in the TOC. The lung cancer model showed intermediate levels of immune infiltration, aligning with the heterogeneous immune landscape commonly observed in non-small cell lung cancer.[35] These results support the potential of the TOC system for modeling tumor-specific immune behavior across multiple cancer types.

Our study also underscores the importance of administration route in drug testing. Drugs delivered via the microfluidic channel, simulating vascular kinetics, resulted in reduced cytotoxic effects in a subset of the analyzed organoid models compared to static application. This indicates that flow-mediated resistance may be a heterogeneous phenomenon dependent on patient-specific tumor characteristics. This resistance was further amplified when drugs had to transverse the endothelial barrier, highlighting the crucial role of vascular architecture and dynamics in therapeutic efficacy. As typical tumor vasculature is considered leaky, HUVEC cells used in our experiments can be considered a potential limitation; however, as they represent the most widely used and well-characterized source of endothelial cells, we selected them for initial validation studies. Incorporation of patient-specific endothelial cells could further enhance the physiological relevance of the model.

In this study, we demonstrated that although both static cultures and the TOC system detect drug-induced decreases in cell viability with increasing drug concentrations, the response was more linear in the TOC than in static cultures. This improved linearity may be advantageous for adjusting patient-specific treatment regimens and reducing the risk of toxicity-related adverse effects. This observation was supported by a retrospective co-clinical analysis using pancreatic cancer organoids. We found that drug-resistant phenotypes in organoids derived from patients with poor clinical outcomes were preserved in the TOC system and mirrored the corresponding clinical trajectories. Conversely, organoids from patients with favorable clinical outcomes exhibited stronger responses to the administered drugs. Together, these findings suggest that the TOC platform may provide complementary information on chemotherapy responses by modeling drug delivery constraints that are absent in conventional static cultures.

Given that more than 90% of oncology drugs fail in clinical trials, improved preclinical models are urgently needed. By integrating vascular, immune, and tumor-specific features, the TOCs may help bridge this translational gap, supporting identification of effective therapies and potentially reducing late-stage failures. While tumor organoids and PDX models have shown partial correlation with clinical outcome,[36,37] to our knowledge this is the first study to evaluate the clinical predictivity of a microfluidic TOC system. Nonetheless, larger-scale validation is required to further confirm its predictive utility.

The open design of the TOC also enables long-term drug resistance studies and molecular profiling of the treatment-surviving cells. ScRNA-seq revealed distinct transcriptional profiles driven by the culture environment, with major changes in metabolic, inflammatory, and drug resistance-related signaling. These findings are especially relevant in light of increasing interest in targeting oxidative phosphorylation and antioxidant defense mechanisms in resistant cancer cells.[28,38] In accordance, the differential transcription factor activity observed between static and fluidic systems, including differential YAP activity, could significantly influence *in vitro* drug testing outcomes. Results obtained using the TOC system can be considered as starting point for further mechanistic and functional assays aimed at validating molecular signatures linked to drug resistance.

Limitations of the study

While the TOC platform offers several key advantages, limitations remain. The design of the current system enables 32 parallel assays on a standard 96-well plate and requires approximately 2×10^6 cells per plate, described as "semi-high throughput." Although the well size is not limiting with surgical samples or organoids, it can be limited when working with small biopsies. Nevertheless, this capacity already allows us to evaluate multiple treatment conditions per patient. Furthermore, ongoing work is focused on the possibility to incorporate the TEER adapter for longitudinal readouts and on validating a 384-well plate format, which would expand the assay to 128 parallel tests while requiring fewer cells per condition. In addition, constant advancement in high-content imaging

systems and automated workflow development will further enhance throughput and reproducibility.

As with all patient-derived tumor models, intra-tumoral heterogeneity may be incompletely represented due to sampling constraints. However, organoids were generated from intact tissue fragments without filtering or enrichment, and prior sequencing data indicate preservation of the major mutational features of the original tumors.[39] Additional sampling strategies may be required to better capture tumor- and metastasis-wide heterogeneity in the future.

We acknowledge that the current implementation is optimized for short- to mid-term assays and does not yet recapitulate chronic tumor-immune co-evolution, long-term immune exhaustion, or matrix remodeling processes observed *in vivo*. Potential long-term complications such as fibrotic matrix deposition, cytokine-driven feedback loops, and progressive immune dysfunction represent important future directions. Incorporation of stromal components and longitudinal cytokine profiling will be required to assess these phenomena and improve translational relevance. While some experiments were conducted without an endothelial barrier to simplify initial readouts of tumor viability, previous work demonstrated that endothelial inclusion significantly modulates drug response. Importantly, even in the absence of endothelium, the TOC architecture alone was sufficient to reveal differences compared to conventional cultures.

Another limitation of the current study is the relatively small number of tumor models analyzed across different cancer types. While the observed differences between models highlight the ability of the TOC system to capture variability in drug responses under different delivery conditions, the cohort size is insufficient to determine whether these effects reflect tumor type-specific characteristics. Future studies including larger numbers of samples across multiple tumor types, as well as incorporation of stromal and immune components, will be necessary to systematically address this question and further evaluate the generalizability of the platform. Nevertheless, the TOC's capacity to uncover drug resistance mechanisms and patient-specific response patterns offers valuable insights into drug action and holds promise for improving personalized cancer therapy.

RESOURCE AVAILABILITY

Lead contact

- Requests for further information and resources should be directed to and will be fulfilled by the lead contact, Heidi M. Haikala (heidi.haikala@helsinki.fi).

Materials availability

- This study did not generate new, unique reagents.

Data and code availability

- ScRNA-seq data are available at the European Genome-phenome Archive (EGA: https://ega-archive.org) under accession number EGAD50000002298. Microscopy data reported in this paper will be shared by the lead contact upon request.
- This paper does not report original code.
- Any additional information required to reanalyze the data reported in this paper is available from the lead contact upon request.

ACKNOWLEDGMENTS

We would like to thank Biomedicum Imaging Unit (BIU), FIMM Single-Cell Analytics core facility (University of Helsinki), and the facilities and expertise of the Drug Discovery, Chemical Biology Consortium (DDCB) unit at the Faculty of Pharmacy, University of Helsinki, supported by HiLIFE and Biocenter Finland. We appreciate the collaboration and support received from Stephen Full, Adyary Fallarero, and Andrew Snyder from Thermo Fisher Scientific regarding the Invitrogen EVOS M3000 Cell Imaging System. We would like to thank Linh Lin (University of Helsinki) for helping with single-cell sample preparations. This study was funded by The Finnish Research Impact Foundation, Research Council of Finland, Business Finland, Sigrid Jusélius Foundation, Tampere Tuberculosis Foundation, Nummela Foundation, Instrumentarium Science Foundation, Finnish Cultural Foundation, Magnus Ehrnrooth Foundation, and Cancer Foundation Finland. Open access was funded by the Helsinki University Library.

AUTHOR CONTRIBUTIONS

E.K., S.M., and H.M.H. designed the study, analyzed and provided data, wrote the manuscript, and provided funding; J.S. analyzed and provided data; M.P., M.G., M.F., G.A., P.J., L.L., S.J., T.P., M.V., K.B., B.A., and L.L. performed experiments and analyzed the data; T.S., E.S., H.K.M., H.S., and I.I. provided clinical data and patient samples; and J.K., A.P., T.N., V.C., R.R., S.J., and P.S. helped with conceptualization and methodology.

DECLARATION OF INTERESTS

M.P., A.P., P.J., J.K., T.N., S.M., and P.S. are employed by Finnadvance Ltd, where P.S. is a founder and shareholder. R.R. and S.J. are employed by Orion, which has been funding part of the scRNA-seq conducted in the study. H.M.H. is a founder and shareholder in precision oncology company, Solid IO, and has been working as a Medical Advisor for Amgen AB.

STAR★METHODS

Detailed methods are provided in the online version of this paper and include the following:

- KEY RESOURCES TABLE
- EXPERIMENTAL MODEL AND STUDY PARTICIPANT DETAILS
  - Patient samples
  - Cell lines
- METHOD DETAILS
  - Study design
  - Cancer organoid establishment and culture
  - PBMC isolation
  - Cell loading to microfluidic chips
  - Permeability assay
  - Immunofluorescence staining of endothelial barriers
  - Drug testing in TOC
  - Drug testing using standard culturing conditions
  - Transmigration assay using tumor and immune cells
  - Live/dead viability assay
  - Sample preparation for scRNA-seq
- QUANTIFICATION AND STATISTICAL ANALYSIS
  - Imaging of endothelial barriers and data reduction
  - Fluorescent microscopy imaging of fixed organoids
  - Image analysis
  - Cell Ranger analysis for scRNA-seq data
  - Quality control of scRNA-seq data
  - Sample demultiplexing of scRNA-seq data
  - Gene set enrichment analysis
  - Transcription factor activity analysis
  - Analysis of pharmacologically active targetable genes
  - Analysis of YAP1/TAZ-TEAD signaling in scRNA-seq
  - Statistics

SUPPLEMENTAL INFORMATION

Supplemental information can be found online at https://doi.org/10.1016/j.crmeth.2026.101475.

Received: December 19, 2025
Revised: March 6, 2026
Accepted: May 5, 2026
Published: June 4, 2026

REFERENCES

1. Mroz, E.A., and Rocco, J.W. (2017). The challenges of tumor genetic diversity. Cancer *123*, 917–927. https://doi.org/10.1002/cncr.30430.
2. Yang, Q., Li, M., Yang, X., Xiao, Z., Tong, X., Tuerdi, A., Li, S., and Lei, L. (2023). Flourishing tumor organoids: History, emerging technology, and application. Bioeng. Transl. Med. *8*, e10559. https://doi.org/10.1002/btm2.10559.
3. Ravi, M., Paramesh, V., Kaviya, S.R., Anuradha, E., and Solomon, F.D.P. (2015). 3D cell culture systems: advantages and applications. J. Cell. Physiol. *230*, 16–26. https://doi.org/10.1002/jcp.24683.
4. Brown, K.M., Xue, A., Mittal, A., Samra, J.S., Smith, R., and Hugh, T.J. (2016). Patient-derived xenograft models of colorectal cancer in pre-clinical research: a systematic review. Oncotarget *7*, 66212–66225. https://doi.org/10.18632/oncotarget.11184.
5. Yoshida, G.J. (2020). Applications of patient-derived tumor xenograft models and tumor organoids. J. Hematol. Oncol. *13*, 4. https://doi.org/10.1186/s13045-019-0829-z.
6. van de Wetering, M., Francies, H.E., Francis, J.M., Bounova, G., Iorio, F., Pronk, A., van Houdt, W., van Gorp, J., Taylor-Weiner, A., Kester, L., et al. (2015). Prospective derivation of a Living Organoid Biobank of colorectal cancer patients. Cell *161*, 933–945. https://doi.org/10.1016/j.cell.2015.03.053.
7. Harada, K., and Sakamoto, N. (2022). Cancer organoid applications to investigate chemotherapy resistance. Front. Mol. Biosci. 9, 1067207.
8. Phan, D.T.T., Wang, X., Craver, B.M., Sobrino, A., Zhao, D., Chen, J.C., Lee, L.Y.N., George, S.C., Lee, A.P., and Hughes, C.C.W. (2017). A vascularized and perfused organ-on-a-chip platform for large-scale drug screening applications. Lab Chip *17*, 511–520. https://doi.org/10.1039/c6lc01422d.
9. Cucullo, L., Hossain, M., Puvenna, V., Marchi, N., and Janigro, D. (2011). The role of shear stress in Blood-Brain Barrier endothelial physiology. BMC Neurosci. *12*, 40. https://doi.org/10.1186/1471-2202-12-40.
10. Shenoy, A.K., and Lu, J. (2016). Cancer cells remodel themselves and vasculature to overcome the endothelial barrier. Cancer Lett. *380*, 534–544. https://doi.org/10.1016/j.canlet.2014.10.031.
11. Li, W., Zhou, Z., Zhou, X., Khoo, B.L., Gunawan, R., Chin, Y.R., Zhang, L., Yi, C., Guan, X., and Yang, M. (2023). 3D Biomimetic Models to Reconstitute Tumor Microenvironment In Vitro: Spheroids, Organoids, and Tumor-on-a-Chip. Adv. Healthcare Mater. *12*, 2202609. https://doi.org/10.1002/adhm.202202609.
12. Zhang, J., Tavakoli, H., Ma, L., Li, X., Han, L., and Li, X. (2022). Immunotherapy discovery on tumor organoid-on-a-chip platforms that recapitulate the tumor microenvironment. Adv. Drug Deliv. Rev. *187*, 114365. https://doi.org/10.1016/j.addr.2022.114365.
13. Chistiakov, D.A., Orekhov, A.N., and Bobryshev, Y.V. (2017). Effects of shear stress on endothelial cells: go with the flow. Acta Physiol. *219*, 382–408. https://doi.org/10.1111/apha.12725.
14. Ma, H.L., Urbaczek, A.C., Zeferino Ribeiro de Souza, F., Bernal, C., Rodrigues Perussi, J., and Carrilho, E. (2023). Replicating endothelial shear stress in organ-on-a-chip for predictive hypericin photodynamic efficiency. Int. J. Pharm. *634*, 122629. https://doi.org/10.1016/j.ijpharm.2023.122629.
15. McQueen, A., and Warboys, C.M. (2023). Mechanosignalling pathways that regulate endothelial barrier function. Curr. Opin. Cell Biol. *84*, 102213. https://doi.org/10.1016/j.ceb.2023.102213.
16. Wettschureck, N., Strilic, B., and Offermanns, S. (2019). Passing the Vascular Barrier: Endothelial Signaling Processes Controlling Extravasation. Physiol. Rev. *99*, 1467–1525. https://doi.org/10.1152/physrev.00037.2018.
17. Nguyen, H.-T., Rissanen, S.-L., Peltokangas, M., Laakkonen, T., Kettunen, J., Barthod, L., Sivakumar, R., Palojärvi, A., Junttila, P., Talvitie, J., et al. (2024). Highly scalable and standardized organ-on-chip platform with TEER for biological barrier modeling. Tissue Barriers *12*, 2315702. https://doi.org/10.1080/21688370.2024.2315702.
18. Peter, M., and Kühnel, F. (2020). Oncolytic Adenovirus in Cancer Immunotherapy. Cancers *12*, 3354. https://doi.org/10.3390/cancers12113354.
19. Nadafi, R., Dong, W., and van Beusechem, V.W. (2025). Immunological Impact of Oncolytic Adenoviruses On Cancer Therapy: Clinical Insights. Eur. J. Immunol. *55*, e70024. https://doi.org/10.1002/eji.70024.
20. Joshi, S. (2024). 8 Promising Adenovirus-associated Oncolytic Virus Therapies. DelveInsight Bus. Res. https://www.delveinsight.com/blog/8-promising-adenovirus-associated-oncolytic-virus-therapies.
21. Feodoroff, M., Hamdan, F., Antignani, G., Feola, S., Fusciello, M., Russo, S., Chiaro, J., Välimäki, K., Pellinen, T., Branca, R.M., et al. (2024). Enhancing T-cell recruitment in renal cell carcinoma with cytokine-armed adenoviruses. Oncoimmunology *13*, 2407532. https://doi.org/10.1080/2162402X.2024.2407532.
22. Nicolson, N.G., Tandurella, J.A., Wu, L.W., Patel, J., Morris, E., Seppälä, T.T., Guinn, S., Zlomke, H., Shubert, C.R., Lafaro, K.J., et al. (2024). Patient-derived Organoid Pharmacotyping As A Predictive Tool for Therapeutic Selection in Pancreatic Ductal Adenocarcinoma. Ann. Surg. *283*, e8–e18. https://doi.org/10.1097/SLA.0000000000006517.
23. Ren, X., Chen, W., Yang, Q., Li, X., and Xu, L. (2022). Patient-derived cancer organoids for drug screening: Basic technology and clinical application. J. Gastroenterol. Hepatol. *37*, 1446–1454. https://doi.org/10.1111/jgh.15930.
24. Seppälä, T.T., Zimmerman, J.W., Suri, R., Zlomke, H., Ivey, G.D., Szabolcs, A., Shubert, C.R., Cameron, J.L., Burns, W.R., Lafaro, K.J., et al. (2022). Precision Medicine in Pancreatic Cancer: Patient-Derived Organoid Pharmacotyping Is a Predictive Biomarker of Clinical Treatment Response. Clin. Cancer Res. Off. J. Am. Assoc. Cancer Res. *28*, 3296–3307. https://doi.org/10.1158/1078-0432.CCR-21-4165.
25. Verduin, M., Hoeben, A., De Ruysscher, D., and Vooijs, M. (2021). Patient-Derived Cancer Organoids as Predictors of Treatment Response. Front. Oncol. *11*, 641980. https://doi.org/10.3389/fonc.2021.641980.
26. Liston, D.R., and Davis, M. (2017). Clinically relevant concentrations of anticancer drugs: A guide for nonclinical studies. Clin. Cancer Res. *23*, 3489–3498. https://doi.org/10.1158/1078-0432.CCR-16-3083.
27. Geyer, M., Schreyer, D., Gaul, L.-M., Pfeffer, S., Pilarsky, C., and Queiroz, K. (2023). A microfluidic-based PDAC organoid system reveals the impact of hypoxia in response to treatment. Cell Death Discov. *9*, 20. https://doi.org/10.1038/s41420-023-01334-z.
28. Bae, T., Hallis, S.P., and Kwak, M.-K. (2024). Hypoxia, oxidative stress, and the interplay of HIFs and NRF2 signaling in cancer. Exp. Mol. Med. *56*, 501–514. https://doi.org/10.1038/s12276-024-01180-8.
29. Shen, X., Zhang, Y., Xu, Z., Gao, H., Feng, W., Li, W., Miao, Y., Xu, Z., Zong, Y., Zhao, J., and Lu, A. (2022). KLF5 inhibition overcomes oxaliplatin resistance in patient-derived colorectal cancer organoids by restoring apoptotic response. Cell Death Dis. *13*, 303. https://doi.org/10.1038/s41419-022-04773-1.
30. Mokhtari, R.B., Ashayeri, N., Baghaie, L., Sambi, M., Satari, K., Baluch, N., Bosykh, D.A., Szewczuk, M.R., and Chakraborty, S. (2023). The Hippo Pathway Effectors YAP/TAZ-TEAD Oncoproteins as Emerging Therapeutic Targets in the Tumor Microenvironment. Cancers *15*, 3468. https://doi.org/10.3390/cancers15133468.

31. Lee, H.J., Diaz, M.F., Price, K.M., Ozuna, J.A., Zhang, S., Sevick-Muraca, E.M., Hagan, J.P., and Wenzel, P.L. (2017). Fluid shear stress activates YAP1 to promote cancer cell motility. Nat. Commun. *8*, 14122. https://doi.org/10.1038/ncomms14122.

32. Bierbaumer, L., Katschnig, A.M., Radic-Sarikas, B., Kauer, M.O., Petro, J.A., Högler, S., Gurnhofer, E., Pedot, G., Schäfer, B.W., Schwentner, R., et al. (2021). YAP/TAZ inhibition reduces metastatic potential of Ewing sarcoma cells. Oncogenesis *10*, 2. https://doi.org/10.1038/s41389-020-00294-8.

33. Banerjee, K., Kumar, S., Ross, K.A., Gautam, S., Poelaert, B., Nasser, M.W., Aithal, A., Bhatia, R., Wannemuehler, M.J., Narasimhan, B., et al. (2018). Emerging trends in the immunotherapy of pancreatic cancer. Cancer Lett. *417*, 35–46. https://doi.org/10.1016/j.canlet.2017.12.012.

34. Banerji, S., Meyers, D.E., Harlos, C., and Dawe, D.E. (2021). The Role of Immunotherapy in the Treatment of Malignant Pleural Mesothelioma. Curr. Oncol. *28*, 4542–4551. https://doi.org/10.3390/curroncol28060385.

35. Lahiri, A., Maji, A., Potdar, P.D., Singh, N., Parikh, P., Bisht, B., Mukherjee, A., and Paul, M.K. (2023). Lung cancer immunotherapy: progress, pitfalls, and promises. Mol. Cancer *22*, 40. https://doi.org/10.1186/s12943-023-01740-y.

36. Ong, L.J.Y., Chia, S., Wong, S.Q.R., Zhang, X., Chua, H., Loo, J.M., Chua, W.Y., Chua, C., Tan, E., Hentze, H., et al. (2022). A comparative study of tumour-on-chip models with patient-derived xenografts for predicting chemotherapy efficacy in colorectal cancer patients. Front. Bioeng. Biotechnol. *10*, 952726. https://doi.org/10.3389/fbioe.2022.952726.

37. Wensink, G.E., Elias, S.G., Mullenders, J., Koopman, M., Boj, S.F., Kranenburg, O.W., and Roodhart, J.M.L. (2021). Patient-derived organoids as a predictive biomarker for treatment response in cancer patients. npj Precis. Oncol. *5*, 30. https://doi.org/10.1038/s41698-021-00168-1.

38. Zhao, Z., Mei, Y., Wang, Z., and He, W. (2022). The Effect of Oxidative Phosphorylation on Cancer Drug Resistance. Cancers *15*, 62. https://doi.org/10.3390/cancers15010062.

39. Alsaed, B., Smolander, J., Laitinen, H., Lin, L., Bobik, N., Lahtinen, L., Räsänen, M., Jansouz, S., Peltonen, K., Jokinen, E., et al. (2024). Ex vivo modeling of precision immuno-oncology responses in lung cancer. Sci. Adv. *10*, eadq6830. https://doi.org/10.1126/sciadv.adq6830.

40. Hamdan, F., Martins, B., Feodoroff, M., Giannoula, Y., Feola, S., Fusciello, M., Chiaro, J., Antignani, G., Grönholm, M., Ylösmäki, E., and Cerullo, V. (2021). GAMER-Ad: a novel and rapid method for generating recombinant adenoviruses. Mol. Ther. Methods Clin. Dev. *20*, 625–634. https://doi.org/10.1016/j.omtm.2021.01.014.

41. van der Walt, S., Schönberger, J.L., Nunez-Iglesias, J., Boulogne, F., Warner, J.D., Yager, N., Gouillart, E., and Yu, T.; scikit-image contributors (2014). scikit-image: image processing in Python. PeerJ *2*, e453. https://doi.org/10.7717/peerj.453.

42. Hao, Y., Stuart, T., Kowalski, M.H., Choudhary, S., Hoffman, P., Hartman, A., Srivastava, A., Molla, G., Madad, S., Fernandez-Granda, C., and Satija, R. (2024). Dictionary learning for integrative, multimodal and scalable single-cell analysis. Nat. Biotechnol. *42*, 293–304. https://doi.org/10.1038/s41587-023-01767-y.

43. Subramanian, A., Tamayo, P., Mootha, V.K., Mukherjee, S., Ebert, B.L., Gillette, M.A., Paulovich, A., Pomeroy, S.L., Golub, T.R., Lander, E.S., and Mesirov, J.P. (2005). Gene set enrichment analysis: A knowledge-based approach for interpreting genome-wide expression profiles. Proc. Natl. Acad. Sci. *102*, 15545–15550. https://doi.org/10.1073/pnas.0506580102.

44. Van de Sande, B., Flerin, C., Davie, K., De Waegeneer, M., Hulselmans, G., Aibar, S., Seurinck, R., Saelens, W., Cannoodt, R., Rouchon, Q., et al. (2020). A scalable SCENIC workflow for single-cell gene regulatory network analysis. Nat. Protoc. *15*, 2247–2276. https://doi.org/10.1038/s41596-020-0336-2.

45. Korotkevich, G., Sukhov, V., Budin, N., Shpak, B., Artyomov, M.N., and Sergushichev, A. (2021). Fast gene set enrichment analysis. bioRxiv. https://doi.org/10.1101/060012.

46. McInnes, L., Healy, J., Saul, N., and Großberger, L. (2018). UMAP: Uniform Manifold Approximation and Projection. J. Open Source Softw. *3*, 861. https://doi.org/10.21105/joss.00861.

47. Blondel, V.D., Guillaume, J.-L., Lambiotte, R., and Lefebvre, E. (2008). Fast unfolding of communities in large networks. J. Stat. Mech. *2008*, P10008. https://doi.org/10.1088/1742-5468/2008/10/P10008.

48. Liberzon, A., Birger, C., Thorvaldsdóttir, H., Ghandi, M., Mesirov, J.P., and Tamayo, P. (2015). The Molecular Signatures Database (MSigDB) hallmark gene set collection. Cell Syst. *1*, 417–425. https://doi.org/10.1016/j.cels.2015.12.004.

49. Wang, Y., Xu, X., Maglic, D., Dill, M.T., Mojumdar, K., Ng, P.K.-S., Jeong, K.J., Tsang, Y.H., Moreno, D., Bhavana, V.H., et al. (2018). Comprehensive Molecular Characterization of the Hippo Signaling Pathway in Cancer. Cell Rep. *25*, 1304–1317.e5. https://doi.org/10.1016/j.celrep.2018.10.001.

50. Pham, T.H., Hagenbeek, T.J., Lee, H.-J., Li, J., Rose, C.M., Lin, E., Yu, M., Martin, S.E., Piskol, R., Lacap, J.A., et al. (2021). Machine-Learning and Chemicogenomics Approach Defines and Predicts Cross-Talk of Hippo and MAPK Pathways. Cancer Discov. *11*, 778–793. https://doi.org/10.1158/2159-8290.CD-20-0706.

STAR★METHODS

KEY RESOURCES TABLE

REAGENT or RESOURCE	SOURCE	IDENTIFIER
Antibodies		
Mouse monoclonal VCAM1	Fisher Scientific/ Invitrogen	Cat# 11314453; Supplier #: MA5-11447; RRID: AB_10979792
Rabbit monoclonal VE-Cadherin	Cell Signaling Technology	Cat# 2500; RRID: AB_10839118
Goat anti-mouse IgG CF488A	Biotium	Cat# 20302; https://biotium.com/product/goat-anti-mouse-igg-hl-highly-cross-adsorbed-min-x-rat/
Donkey anti-rabbit IgG CF594	Biotium	Cat# 20152; RRID: AB_10563030
DAPI	Fisher Scientific/ Invitrogen	Cat# 10116287; Supplier#: 62248
Cy5.5-EpCam	Biolegend	Cat# 324214; RRID: AB_2098808
PE/Cy7-CD3	BD Biosciences	Cat# 557851; RRID: AB_396896
TotalSeq -A0251 anti-human Hashtag 1 Antibody	BioLegend	Cat# 394601; RRID: AB_2750015
TotalSeq -A0252 anti-human Hashtag 2 Antibody	BioLegend	Cat# 394603; RRID: AB_2750016
TotalSeq -A0253 anti-human Hashtag 3 Antibody	BioLegend	Cat# 394605; RRID: AB_2750017
TotalSeq -A0254 anti-human Hashtag 4 Antibody	BioLegend	Cat# 394607; RRID: AB_2750018
Bacterial and virus strains		
AdΔ245/3	Hamdan et al.[40]	N/A
AdΔ245/3-CXCL10	Hamdan et al.[40]	N/A
Biological samples		
Lung cancer organoids (ORG2) made from patient tumor tissue	Living biobank; HaikaLab	ORG2
Lung cancer organoids (ORG55) made from patient tumor tissue	Living biobank; HaikaLab	ORG55
Lung cancer organoids (ORG67) made from patient tumor tissue	Living biobank; HaikaLab	ORG67
Lung cancer organoids (Lung27) made from patient tumor tissue	Living biobank; HaikaLab	Lung27
Lung cancer organoids (ORG96T) made from patient tumor tissue	Living biobank; HaikaLab	ORG96T
Pancreatic cancer organoids (PO109T) made from patient tumor tissue	Living biobank; Seppänen lab	PO109T
Pancreatic cancer organoids (PO117T) made from patient tumor tissue	Living biobank; Seppänen lab	PO117T
Pancreatic cancer organoids (PO139T) made from patient tumor tissue	Living biobank; Seppänen lab	PO139T
Pancreatic cancer organoids (PO126T) made from patient tumor tissue	Living biobank; Seppänen lab	PO126T
Pancreatic cancer organoids (PO121T) made from patient tumor tissue	Living biobank; Seppänen lab	PO121T
Mesothelioma cancer organoids (Meso15) made from patient tumor tissue	Living biobank; HaikaLab	Meso15
Patient PBMCs	HUS, Helsinki University Hospital	N/A

(Continued on next page)

CellPress
OPEN ACCESS

Continued

REAGENT or RESOURCE	SOURCE	IDENTIFIER
Chemicals, peptides, and recombinant proteins		
1x RBC lysis buffer	BioLegend	Cat# 420302
Martigel, growth factor reduced basement membrane matrix, phenol red-free, LDEV-free	Corning	Cat# 356231
TrypLE Express	Gibco	Cat# 12604-054
Endothelial Cell Growth Medium 2, ready-to-use kit	Sigma-Aldrich	Cat# C-22011
ImmunoCult CD3/CD28/CD2 T cell activator solution	StemCell Technologies	Cat# 10970
Live stain Celltracker Violet BMQC dye	Thermo Fisher Scientific	Cat# C10094
Live stain Celltracker Orange CMRA dye	Thermo Fisher Scientific	Cat# C34551
70 kDA TRITC-dextran	TdB Labs	Cat# TD70
4 kDa FITC-dextran	TdB Labs	CAS# 60842-46-8; Cat# FD4
TNF-α	Thermo Fisher Scientific	Cat# 10304263
IFN-γ	Thermo Fisher Scientific	Cat# 10474733
Triton X-100	Thermo Fisher Scientific	Cat# BP151-100
Tween 20	Thermo Fisher Scientific	Cat# BP337-100
BSA	Biowest	Cat# A0296
Dispase II solution	Gibco	Cat# 17105-041
CellTracker Green CMFDA dye	Thermo Fisher Scientific	Cat# C2925
Calcein-AM	Thermo Fisher Scientific	Cat# 65-0853-78
Propidium Iodide, PI	Thermo Fisher Scientific	Cat# 11599296
Zinc-formalin fixative	Sigma Aldrich	Cat# Z2902
Human TruStain FcX blocking reagent	BioLegend	Cat# 422302
Gemcitabine Hydrochloride	Selleck Biotechnology GmbH	Cat# S1149; CAS# 122111-03-9
Carboplatin	Sigma Aldrich	Cat# C2538; CAS# 41575-94-4
Pemetrexed disodium heptahydrate	Sigma Aldrich	Cat# SML1490; CAS# 357166-29-1
Critical commercial assays		
CellTiter-Glo Luminescent cell viability assay	Promega	Cat# G7571
10x Genomics Chromium Single Cell 3′ Reagent kits	10x Genomics	CG000317
Deposited data		
sensitive ScRNA-seq data	This paper	European Genome-phenome archive (EGA); https://ega-archive.org; EGAD50000002298
Raw microscopy data	This paper	shared upon request
Experimental models: cell lines		
HUVEC	Lonza Bioscience	Cat# C2519A
HIMEC	CellBiologics	Cat# H-6203
HLMVEC	CellBiologics	Cat# H-6011
Software and algorithms		
COMSOL Multiphysics	Nguyen et al.[17]	https://www.comsol.com/comsol-multiphysics; RRID:SCR_014767
CellProfiler Image Analysis Software (4.2.6)	CellProfiler™ cell image analysis software	https://cellprofiler.org; PRID:SCR_007358
Scikit-image (0.17)	van der Walt et al.[41]	https://scikit-image.org/; RRID:SCR_021142
FlowJo 10.8.1	FlowJo, BD	https://www.flowjo.com/solutions/flowjo; RRID:SCR_008520

(*Continued on next page*)

Continued		
REAGENT or RESOURCE	SOURCE	IDENTIFIER
Fiji-ImageJ	Fiji	https://hpc.nih.gov/apps/Fiji.html; RRID:SCR_002285
GraphPad Prism 9	GraphPad	https://www.graphpad.com; RRID:SCR_002798
Cell Ranger (v8.0.0)	10x Genomics	https://www.10xgenomics.com/support/software/cell-ranger/8.0; RRID:SCR_017344
Seurat v5.0.1	Hao et al.[42]	https://satijalab.org/seurat/articles/install.html; RRID:SCR_016341
Gene Set Enrichment Analysis (GSEA) algorithm	Subramanian et al.[43]	RRID:SCR_003199
pySCENIC v0.12.1	Van de Sande et al.[44]	https://scenic.aertslab.org/; RRID:SCR_025802
fgsea R package (v1.24.0)	Korotkevich et al.[45]	RRID:SCR_020938
R package v3.4.4	R Project for Statistical Computing	https://www.r-project.org/; RRID:SCR_001905
Other		
AKITA Plate96-Single, PET-membrane 10 μm pore size	Finnadvance Oy	ref:01061, 32 independent assays

EXPERIMENTAL MODEL AND STUDY PARTICIPANT DETAILS

Patient samples

Patient primary tumor and blood samples were collected following the Declaration of Helsinki from consented lung and pancreatic cancer patients with concurrent BioBank deposits. Helsinki University Hospital IRB has been granted all required permits (HUS/237/2021 and HUS/2761/2021) with the statement from the ethical board (HUS/970/2021). Peripheral blood was collected into lithium heparin vacuum tubes according to Helsinki University Hospital Laboratory's (HUSLAB) standard protocols during a routine laboratory visit.

Cell lines

The following endothelial cell lines were used in the study, human umbilical vein endothelial cells (HUVECs from pooled donors, Lonza Bioscience), human intestinal microvascular endothelial cells (HIMECs, CellBiologics) and human lung microvascular endothelial cells (HLMVECs, CellBiologics). All cell lines were cultured in +37°C, 5% CO_2 in Endothelial Cell Growth Medium 2 consisted of Basal media and Supplement Mix (Basal media and Supplement Mix ready-to-use kit, cat. # C-22011 from Sigma-Aldrich) in the presence of 10% FBS and 1x penicillin/streptomycin (100x solution contains 5000 units/mL of both penicillin and streptomycin, ThermoFisher Scientific) using Nunc EasYFlask cell culture treated flasks (cat. # 156499, ThermoFisher Scientific). According to manufacturers' recommendations, additional gelatin coating was used for culturing HIMECs and HLMVECs. Endothelial cells were passaged upon reaching 80–90% confluency and medium was changed twice a week.

METHOD DETAILS

Study design

The primary objective of this study was to evaluate the benefits and applicability of the microfluidic TOC platform for predicting patient-specific responses to chemotherapy treatments. To achieve this, we compared experimental results obtained using traditional 384-well or 96-well plates ("static") with those derived from the TOC platform. Additionally, we validated our findings by comparing them with patient clinical records in collaboration with Helsinki University Hospital (HUS), which allowed us to assess the relevance and potential clinical applications of our results.

We tested the sensitivity of the developed workflows by applying them to three cancer types: pancreatic ductal adenocarcinoma (PDAC), lung adenocarcinoma, and malignant pleural mesothelioma. The experimental tasks were designed to assess cancer cell viability and resistance development in response to chemotherapy treatments. Fluorescence microscopy imaging and scRNA-seq were used to evaluate cell viability and gene expression profiles. For experiments analyzing immune cell migration, we studied the enrichment of PBMCs at the tumor site within the open-top chamber containing cancer organoids. Immune cell migration was monitored via fluorescence microscopy over a 72-h period.

The number of independent replicates varied from 3 to 5, depending on the experimental setup. The exact number of replicates for each experiment is specified in the corresponding figure legends.

Cancer organoid establishment and culture

Patient tumor tissue samples were delivered from HUS in a wash medium within one hour after surgery and immediately processed according to the following protocol.[39] Intact tissue fragments were minced with surgical scalpels into pieces and further dissociated overnight with collagenase solution in 10 mL of DMEM-medium supplemented with 1% Pen/Strep, 1% L-glutamine and 0.02% collagenase. Tissue digestion was stopped with 10% v/v fetal bovine serum (FBS) in phosphate buffered saline (PBS), centrifuged for 5 min at 400 x g, and then washed several times with PBS. For bloody samples, lysis of red blood cells was performed using 2 mL of 1x RBC lysis buffer (cat. # 420302, BioLegend) diluted in ultra-pure (Milli-Q) water before PBS-washes. Isolated tumor cells were embedded in 100% Matrigel (cat. # 356231, Corning) for self-assembly into 3D structures and supplemented with organoid medium after gel solidification. There was no prior filtering or enrichment of certain cell types to preserve intrinsic tumor heterogeneity. Furthermore, prior whole-exome sequencing (WES) analysis has confirmed that organoids preserve the genetic features and genetic diversity of the original patient tumors.[39] Organoid growth was assessed using Nikon Eclipse TS100 brightfield microscope and organoids were passaged once every 1–2 weeks at a 1:2 split ratio.

For passaging, organoids were dissociated using warm TrypLE Express (Gibco, cat. #12604–054) for 10 min at 37°C. Dissociation was terminated with cold 20% FBS in DMEM/F12. The dissociated cells were centrifuged at 300 x g for 5 min at 4°C and washed with cold advanced DMEM/F12. Organoids were either broken into single cells or small aggregates, then resuspended in 100% Matrigel and plated as 30 μL domes in 24-well plates. The plates were incubated at 37°C for 30 min to allow gel solidification. Afterward, the organoids were overlaid with warm special organoid medium.

PBMC isolation

PBMCs were isolated from peripheral blood by SepMate density gradient centrifugation using room temperature Ficoll-Paque density gradient medium according to the manufacturer's instructions. After sample centrifugation at 1200 x g for 10 min at room temperature with breaks on, PBMCs and plasma were poured off into a separate 50 mL tube, washed twice with PBS +2% FBS and resuspended in RPMI-1640 supplemented with 2 mM L-Glutamine, 100 U/mL penicillin, 100 μg/mL streptomycin, and 10% FBS (complete RPMI media). Collected PBMCs were assessed for viability and either cultured in complete RPMI media or cryopreserved for later use. Frozen PBMCs were gently thawed in a 37°C water bath, washed twice with complete RPMI media and left for recovery overnight.

Cell loading to microfluidic chips

Microfluidic experiments were done in the AKITA platform, either in a polydimethylsiloxane (PDMS)-based prototype, polyethylene terephthalate (PET)-membrane 3 and 8 μm pore size (24 independent assays) or fully plastic versions of AKITA Plate96-Single, PET-membrane 10 μm pore size (Finnadvance, ref. 01061, 32 independent assays). Both parts of microfluidic unit were washed several times with PBS and coated with fibronectin solution (50 μg/mL in PBS) overnight. Desired quantities of organoids embedded in 70% Matrigel were loaded to the open-top chamber of each unit and supplemented with special organoid medium[39] after gel solidification. For transmigration experiments, organoids were additionally labeled using live stain (Celltracker Violet BMQC dye, cat. #C10094, Thermo Fisher Scientific) according to manufacturer's protocol before embedding to Matrigel.

In experiments that required an endothelial barrier, appropriate human endothelial cells, HUVECs, HIMECs or HLMVECs, were loaded to the microchannels (100000 cells/channel) in endothelial growth medium before organoids' seeding when a PDMS-based plate was used or after that in case of a plastic microfluidic plate and incubated for at least four hours in an upside-down position to increase attachment of cells to the membrane side. Afterward, the TOC system was placed on the rocker inside a +37°C, 5% CO_2 incubator. Experimental parameters (1 rpm rocking speed, 10-degree tilt with 30 s holds on each side) were selected based on a simulation using finite element analysis in COMSOL Multiphysics described in the previous work.[17] Induced liquid flow was corresponded to approximately 2.5 dyn/cm^2 which is in a range of commonly agreed vascular shear stress. Fresh special organoid or endothelial cell media (Endothelial Cell Growth Medium 2 (Basal media and Supplement Mix ready-to-use kit, cat. # C-22011 from Sigma-Aldrich) supplemented with Penicillin/Streptomycin and 10% FBS) were added to the corresponding parts of the system every other day.

In experiments assessing the transmigration of immune cells in the absence of adenovirus infection of cancer organoids, PBMCs were additionally activated for 2–3 h with ImmunoCult CD3/CD28/CD2 T cell activator solution (cat. # 10970, StemCell Technologies) prior their labeling with live stain Celltracker Orange CMRA dye (cat. #C34551, Thermo Fisher Scientific). In all experiments with immune cells, PBMCs were added to the microchannels of the plate in T cell medium (RPMI-1640 supplemented with 1% P/S, 1% ultraglutamine I and 10% human serum), and incubated on the rocker for 72 h. Images of migrated cells in the upper top chamber were taken either with Leica Thunder wide field fluorescence microscope (Leica Microsystems) or the Invitrogen EVOS M3000 Cell Imaging System (Thermo Scientific).

Permeability assay

Barrier permeability was assessed with fluorescent-labeled molecules, 70 kDa TRITC-dextran (TdB Labs, cat: # TD70) and 4 kDa FITC-dextran (TdB Labs, CAS #: 60842-46-8, cat: # FD4). The open-top chambers and microchannels were washed twice with PBS (Gibco) and once with HBSS (Gibco). 70 μL of fresh HBSS was added to the open-top chambers, and zero-timepoint samples were collected. The HBSS in microchannels was then replaced with 100 μL of dextran solution containing 0.05 mg/mL of both tracers.

The plate was tilted couple of times to ensure even flow throughout the microchannels. Incubation was done on a AKITA Wave rocker for 120 min with 10° tilting angle, 30 s hold time, 1 rpm. After this, samples were collected from open-top chambers and microchannels to a microplate (PerkinElmer). Samples were read with a TECAN Infinite M1000Pro -plate reader using 498 nm excitation and 518 emission wavelengths for 4 kDa and 554 nm excitation and 578 nm emission wavelengths for 70 kDa tracer. Apparent permeability at 120 min was calculated by using the following equation:

$$P_{app} = \frac{V}{A \times [C]_{donor}} \times \frac{d[C]_{receiver}}{dt},$$

where *V is* the open-top chamber (receiver) volume, *A* is the membrane surface area 0.07 cm^2, $[C]_{donor}$ is the initial tracer concentration in microchannels, $d[C]_{receiver}$ is the open-top chamber tracer concentration (ng/mL) and *dt* is the incubation time 120 min.

Immunofluorescence staining of endothelial barriers

Fully plastic AKITA Plate96-Single (Finnadvance) plates with 10 μm pore size were used. For establishment of endothelial barrier HUVEC cells were seeded to microchannels with 70000 cells/channel. HUVEC barriers were cultured for 5 days under constant flow. The cells were subjected to inflammatory cytokines TNF-α (cat. # 10304263, Thermo Fisher Scientific) and IFN-γ (cat. # 10474733, Thermo Fisher Scientific) in increasing concentrations of 0–100 ng/mL for 48 h. After the cytokine treatment the cells were fixed with 4% paraformaldehyde (PFA). For permeabilization, the samples were treated with 0.1% Triton X-100 (cat. # BP151-100, Thermo Fisher Scientific) in PBS for 10 min. To prevent nonspecific binding, the samples were blocked with a blocking buffer containing 1% Tween 20 (cat. # BP337-100, Thermo Fisher Scientific) and 3% BSA (cat. # A0296, Biowest) in PBS for 3 h at room temperature (RT). Primary antibodies VCAM1 mouse monoclonal antibody (cat. # 11314453; Supplier#: MA5-11447, Fisher Scientific) diluted to 1:100 and VE-Cadherin Rabbit monoclonal antibody (cat. # 2500, Cell Signaling Technology) diluted to 1:400 were prepared in blocking buffer and incubated on the samples at +4°C overnight. After the incubation the cells were washed 3 × 10 min with 1% Tween 20 in PBS. Secondary antibodies CF488A Goat Anti-Mouse IgG (cat. # 20302, Biotium) diluted to 1:2000, CF594 Donkey Anti-Rabbit IgG (cat. # 20152, Biotium) diluted to 1:2000 and 4′,6′-diamidino-2-phenylindole (DAPI) (cat. # 10116287, Supplier#:62248, Fisher Scientific) diluted to 1:1000 were prepared in blocking buffer and incubated on the samples at RT for 2 h with gentle rocking. After the incubation, the samples were washed 3 × 10 min and stored in PBS.

Drug testing in TOC

AKITA PDMS-based prototype and fully plastic AKITA Plate96-Single (Finnadvance) microfluidic systems described above were used. Lung tumor organoids were isolated from Matrigel and dissociated into single cells or 3–5 cell aggregates by 10–15 min incubation with warm TrypLE and intensive pipetting every 3–5 min. Pancreatic tumor organoids were additionally treated with Dispase II solution (cat. # 17105–041, Gibco) for 30 min at 37°C and centrifuged before incubation with TrypLE Express. Dissociated tumor cells were resuspended in 70% Matrigel at about 3000 cells/μL and 20 μL of suspension (60000 cells/chamber) was loaded to open-top chambers, 20 min after organoid-gel mixture solidification special organoids medium was slowly added on top of the gel. In chip units that required the presence of an endothelial barrier, 50 μL of HUVECs at concentration 2000 cells/μL were loaded to microchannels before or after seeding tumor cells depending on used type of AKITA plate. TOC plate was incubated under constant rocking at 37°C in a 5% CO_2 incubator for 7 days to allow the formation of tumor organoids and endothelial monolayer. Types and amounts of drugs used in the TOC system for drug testing experiments were selected according to corresponding patient data. In addition to vehicle treatment, three or four drug concentrations were used – clinically relevant one and additional concentrations above and below it. All drugs were added to the TOC system through microchannels and their effect on organoids' viability was assessed 72 h after that.

Drug testing using standard culturing conditions

As a comparison to drug testing in the TOC system, a standard 384-well plate was used. Dissociated tumor organoids were resuspended in 70% Matrigel at 600 cells/μL concentration and 10 μL of this suspension was loaded into each well. Appropriate drugs at varying concentrations were added to the wells 4 days after organoid formation, and the viability of cells were assessed 72 h later.

Transmigration assay using tumor and immune cells

For experiments testing the migration of PBMCs toward oncolytic virus infected tumor cells, the plastic AKITA plate with pore size 10 μm was used. Patient-derived tumor organoids from PDAC, lung adenocarcinoma or mesothelioma were infected with adenoviruses AdΔ245/3 or AdΔ245/3-CXCL10 expressing the chemokine CXCL10[40] at multiplicity of infection (MOI) 50 or left uninfected. After infection, the tumor organoids were embedded into 50% Matrigel and placed on top of the filter. After 12 h, unmatched PBMCs fluorescently labeled with 5 μM CellTracker Green CMFDA Dye (cat. #C2925, Thermo Fisher Scientific) were added to the lower microchannel and incubated on a rocker for 72 h. Images of migrated cells in the upper top chamber were taken at different time points with the Invitrogen EVOS M3000 Cell Imaging System (Thermo Scientific). Migrating immune cells were quantified with Celleste 6.01 software (Thermo Scientific).

For all other migration assays analysis of background immune cell migration using fluorescence microscopy and Flow Cytometry, dissociated non-infected Lung tumor organoids and unmatched PBMCs labeled with live dyes prior loading to TOC system, were

used at 1:4 ratio. First, the number of migrated immune cells was assessed qualitatively with live cell imaging using Leica Thunder widefield microscope (Leica Microsystems) equipped with environmental chamber (37°C, 5% CO_2). The following settings were used during z stack image acquisition – 10× HC PL Fluotar objective, Bright Field (BF), 395 nm and 550 nm fluorescent channels, tile scanning and build in deconvolution protocol for image preprocessing. Second, cells from open-top chambers and corresponding microchannels were extracted from TOC system and analyzed using Flow Cytometry. First, immune cells from microchannels were collected to separate tubes. Second, 20 μL of warm TrypLE Express were added to open-top chambers to soften Matrigel and then cell suspensions were transferred to separate tubes to complete dissociation into single cells. All further steps were performed on ice or at 4°C. Cells from both parts of chip units were centrifuged (400 x g for 5 min at 4°C) and washed twice with cold fluorescent activated cell sorting (FACS) buffer (10% FBS in PBS), aliquots of about 20000 cells were transferred into separate tubes as "unlabeled controls", the rest of cells were labeled with Cy5.5-EpCam (epithelial marker for tumor cells, cat. # 324214, Biolegend) and PE/Cy7-CD3 (marker for CD3-positive lymphocytes, cat. # 557851, BD Biosciences) antibodies. After labeling, cells were fixed with 4% PFA and presence of both cell types in each fraction was assessed using BD FACS-Verse Cell Analyzer (BD Biosciences). Quantitative analysis of immune cell migration was done using FlowJo 10.8.1 software (FlowJo, BD). Results were presented as an averaged percentage of CD3-positive cells registered in microchannels and open-top chambers.

Live/dead viability assay

The viability of organoids was assessed either using CellTiter-Glo Luminescent Cell Viability assay (cat. #G7571, Promega) according to the manufactory instructions or using a combination of live and dead stains, that are Calcein-AM (cell-permeant fluorescent dye that determines viable cells, cat. # 65-0853-78, Thermo Fisher Scientific) and Propidium Iodide (PI, membrane-impermeable fluorescent DNA stain that determines dead cells, cat. # 11599296, Thermo Fisher Scientific). Organoids in open-top chambers of the TOC system or in wells of standard plate were incubated for 30 min at 37°C with staining solution (4 μM Calcein-AM and 5 μg/μL PI in special organoids medium), washed twice with special organoids medium and fixed for 15 min at RT using Zinc-formalin fixative (cat. #Z2902, Sigma Aldrich) diluted in Milli-Q to 1:4 ratio. After fixation samples were washed twice with PBS and were ready for microscopy imaging.

Sample preparation for scRNA-seq

Single-cell suspensions from ORG2 and PO109T models were seeded to standard 96-well or microfluidic plates and cultured for 3 days to form organoid structures. Appropriate chemotherapeutics/vehicles were added on top of organoids or through microchannels for "static" or "microfluidic" conditions, respectively. After being cultured with drugs for 4 and 7 days, cells were collected and dissociated into single cells using TrypLE Express or Dispase II/TrypLE Express for ORG2 and PO109T, respectively, followed by 2–3 washes in 10 mL chilled PBS and divided into groups depending on their viability. Cells were resuspended in 100 μL cold PBS with 10% FBS and blocked for 10 min on ice in the presence of TruStain FcX blocking reagent (FcX, BioLegend, cat. # 422302). A unique TotalSeq-A anti-human hashing antibody (TotalSeq-A0251, TotalSeq-A0252, TotalSeq-A0253, TotalSeq-A0254, BioLegend) was added to each sample (2 μg per sample) and cells were incubated for 30 min on ice. Depending on viability, cells were washed 3–5 times with 3.5 mL washing buffer 1 (0.04% bovine serum albumin (BSA) in PBS when viability is >85%), washing buffer 2 (1% BSA in PBS when viability is 70–85%), or washing buffer 3 (10% FBS in PBS when viability is <70%), then samples were combined in cold PBS +0.04% BSA and proceeded to scRNA-seq.

scRNA-seq was done using 10x Genomics Chromium Single Cell 3′ Reagent Kits with Feature Barcoding technology for Cell Surface Protein (CG000317). The Chromium Single Cell 3′ RNAseq run, and hashtag oligonucleotides (HTO)-library preparation were done using the Chromium Next GEM Single Cell 3′ Gene Expression (version v3.1). The Sample libraries were sequenced on the Illumina NovaSeq 6000 system using read lengths: 28bp (Read 1), 10bp (i7 Index), 10bp (i5 Index) and 90bp (Read 2).

QUANTIFICATION AND STATISTICAL ANALYSIS

Imaging of endothelial barriers and data reduction

After cytokine treatment and antibody staining as described above, cells in the microfluidic chamber were imaged using a Cytation 5 (BioTek) with a 10× Plan Fluorite phase-contrast NA 0.3 objective (Olympus 1320516). Fluorescence imaging was performed using filter cubes for DAPI (1225100) to visualize nuclei, Texas Red (1225102) for Anti-VE-Cadherin, and GFP (1225101) for Anti-VCAM1, capturing a 2 × 2 field of view per well.

Raw images were preprocessed using scikit-image 0.17,[41] applying a rolling ball algorithm for background subtraction. Segmentation was performed based on the nuclear signal (Global Minimum Cross-Entropy), and the mask was expanded to include the anti-VE-Cadherin signal using CellProfiler 4.2.6. The mean antibody signal intensity per segment was used to quantify VCAM1 expression per cell.

To estimate cell roundness, the eccentricity of the expanded anti-VE-Cadherin mask (representing the entire cell) was measured. Eccentricity, defined as the ratio of the distance between the foci of an ellipse and its major axis length, yields a unitless value approaching zero for circular shapes.

Fluorescent microscopy imaging of fixed organoids

Z-stacks of fixed organoids were taken with 10× HC PL Fluotar objective using an inverted fluorescent widefield microscope Leica Thunder (Leica Microsystems). Imaging settings included recording signals using Bright Field (BF), green (470 nm) and red (550 nm) channels, and mosaic coverage of the well's area with 9 tiles. Resulted image sequences were preprocessed using the built-in deconvolution function (''Instant computational clearing – ICC''). The rest of the analysis was done in Fiji-ImageJ software.

Image analysis

Fiji-ImageJ software was used to process images acquired with the Leica Thunder widefield microscope. Z-stacks of images were split into separate channel sequences, which were processed independently. These were BF, an overview of organoids; green channel, live organoids; and red channel, dead ones. Images in each fluorescent channel underwent the same steps, which include I – ''Z Project'' to bring all organoids of the stack in focus (Image, Stacks, Z Project, Max intensity), II – ''LabKit'' plugin for image segmentation via pixel classification to select live or dead organoids (Plugins, Labkit), with following mask creation (Process, Binary, Convert to mask), III – applying mask to the initial image to remove background signal (Process, Image calculator, assign Mask as ''image 1'', initial image as ''image 2'' and proceed using operation ''AND''), IV – setting threshold (Image, Adjust, Threshold, select Otsu method) so that signal from only stained organoids in chosen range could be used in area determination, and V – measuring the area covered by either live or dead organoids (Analyze, Measure (in ''Set Measurements'' select ''Area'' and ''limit to threshold'')). Viability of cells was determined as a ratio between areas covered by stained organoids in green (''live'') and red (''dead'') channels. Each experimental condition was repeated three times, and an average ratio value was calculated for each drug concentration. Results were presented as a percentage of viability plus SD in relation to the ratio measured for organoids treated with a vehicle and plotted against varying drug concentrations. GraphPad Prism 9 was used for graphical data presentation (GraphPad).

Cell Ranger analysis for scRNA-seq data

Raw scRNA-seq data were pre-processed using Cell Ranger count (v8.0.0). The human reference genome (refdata-gex-GRCh38-2024-A) was obtained from the 10x Genomics website.

Quality control of scRNA-seq data

scRNA-seq data quality control and analysis were conducted using Seurat (v5.0.1).[42] Initial filtering employed a relaxed minimum unique molecular identifier (UMI) count threshold of 100 to exclude empty droplets. Cells with more than 25% mitochondrial reads were also removed to exclude low-quality cells. Normalization was performed using the LogNormalize method, followed by selection of the top 2000 highly variable genes via the variance-stabilizing transformation (VST) method. These genes were scaled using the ScaleData function, and dimensionality reduction was achieved with principal component analysis (PCA), retaining the first 30 components. Visualization of the data was carried out using UMAP,[46] and cell clustering was performed with the Louvain graph-based algorithm[47] with a resolution value of 0.5.

Upon cluster analysis, cell populations with characteristics consistent with empty droplets (i.e., low UMI and feature counts) were identified and excluded. The analysis was repeated starting from the selection of highly variable genes, ensuring robust clustering and visualization of the remaining high-quality cells.

Sample demultiplexing of scRNA-seq data

The HTODemux method of Seurat (v5.0.1) was employed to perform sample demultiplexing. The cells classified as doublets or negatives were excluded, and the singlets were included for downstream analysis.

Gene set enrichment analysis

Hallmark gene signatures, representing well-defined biological states or processes, were obtained from the MSigDB database.[48] To analyze pathway enrichment between the microfluidic and static conditions, we compared differential expression (DE) changes between the microfluidic and static conditions only in the control samples. DE analysis was conducted using the Wilcoxon rank-sum test from the FindMarkers function of Seurat, applied separately to the two organoid samples. Genes were ranked based on the product of the log2 fold-change sign and the negative logarithm (base 10) of the nominal *p*-value. This pre-ranked gene list served as input for pathway enrichment analysis using the Gene Set Enrichment Analysis (GSEA) algorithm,[43] implemented in the fgsea R package (v1.24.0).[45]

For the comparison between chemotherapy and control conditions, a similar workflow was followed. However, the analysis was conducted separately for each time point and platform, comparing chemotherapy and control samples.

Transcription factor activity analysis

Transcription factor (TF) activity analysis and gene regulatory network inference were performed using pySCENIC (v0.12.1).[44] The analysis was conducted separately for each sample, each organoid, and for the two organoids combined. To visualize the regulon activity at single cell level, area under the curve (AUC) values were averaged with the arithmetic mean and visualized in a heatmap along with the corresponding TF expression levels.

Analysis of pharmacologically active targetable genes

A list of pharmacologically active targetable genes was acquired from Drugbank (last edit: March 14, 2024). DE analysis was performed between the control samples of the microfluidic and static conditions using the Wilcoxon rank-sum test. The *p*-values were corrected for multiple comparisons using the Benjamini-Hochberg procedure. The log2FC and false discovery rate (FDR) values were visualized as a dotplot using the ggplot2 R package.

The analysis was repeated to examine DE changes in pharmacologically active targetable genes following chemotherapy. A heatmap was used to visualize the power from receiver operating characteristic (ROC) analysis, where power is defined as $|AUC - 0.5| \times 2$. A higher power value indicates better predictive performance of a gene when classifying cells in chemotherapy vs. control.

Analysis of YAP1/TAZ-TEAD signaling in scRNA-seq

The TEAD1(+) regulon activity scores were computed using pySCENIC (v0.12.1). The activity scores (AUC) were averaged with the arithmetic mean, and a log2 fold-change (log2FC) was calculated between the chemotherapy and control conditions for each platform and timepoint. The log2FC values were visualized as a barplot using the ggplot2 (v3.4.4) R package.

The GSEA method from the fgsea R package was employed to investigate the activation of YAP1/TAZ-TEAD signaling following chemotherapy. Two different YAP1/TAZ-TEAD signaling pathways were used (Table S2), which were derived from previous studies.[49,50] Pre-ranked gene lists for GSEA were generated based on the signed nominal *p*-value from the Wilcoxon rank-sum test.

The scaled average expression values of *TEAD1*, *TEAD2*, *TEAD3*, *TEAD4*, *WWTR1* and *YAP1* were visualized using the DotPlot function of Seurat.

Statistics

All statistical tests were performed in GraphPad Prism 9. Initial image processing was done in corresponding microscopy software, and the following analysis was done in Fiji-ImageJ. Statistical significance was evaluated using one-way ANOVA without correction for multiple comparisons since each comparison considered as independent (Fisher's LSD test was used as follow up test). For comparison between two groups unpaired two-tailed *t* test was used. The specific statistical methods used in each experiment are explained in the respective figure legends. All data are presented as means and standard deviation (SD), with specific sample size provided in each figure legend. Levels of statistical significance are denoted as follows: $p < 0.05$ (*), $p < 0.01$ (**), $p < 0.001$ (***), $p < 0.0001$ (****). $p < 0.05$ was considered statistically significant.

Cell Reports Methods, Volume *6*

Supplemental information

Precision tumor-on-chip for personalized assessment of drug efficacy and immune cell delivery

Elena Kremneva, Johannes Smolander, Mimosa Peltokangas, Mikaela Grönholm, Lilja Lahtinen, Shadi Jansouz, Sanna Vainionpää, Bassel Alsaed, Kristian Borenius, Anniina Palojärvi, Pauliina Junttila, Jere Kettunen, Tuomas Pylkkö, Michaela Feodoroff, Gabriella Antignani, Harri K. Mustonen, Markus Vähä-Koskela, Tuan Nguyen, Prateek Singh, Toni T. Seppälä, Eva Sutinen, Reetta Riikonen, Satu Juhila, Vincenzo Cerullo, Hanna Seppänen, Ilkka Ilonen, Sebastien Mosser, and Heidi M. Haikala

DOCUMENT S1
Figures S1–S3

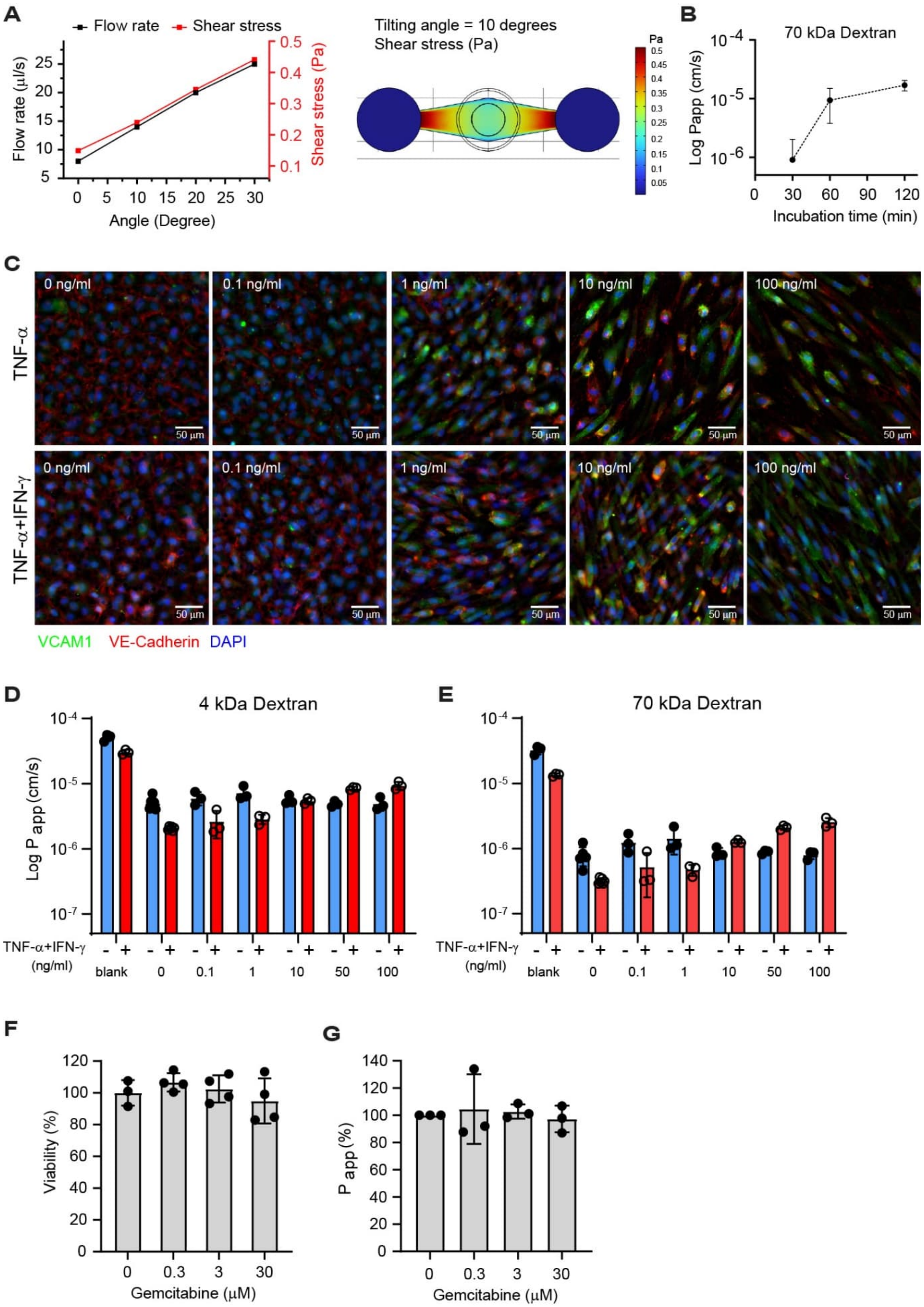
A
Flow rate
Shear stress
Flow rate (µl/s)
Shear stress (Pa)
Angle (Degree)
Tilting angle = 10 degrees
Shear stress (Pa)
Pa
B
70 kDa Dextran
Log Papp (cm/s)
Incubation time (min)
C
TNF-α
TNF-α+IFN-γ
0 ng/ml
0.1 ng/ml
1 ng/ml
10 ng/ml
100 ng/ml
50 µm
VCAM1 VE-Cadherin DAPI
D
4 kDa Dextran
Log P app (cm/s)
TNF-α+IFN-γ (ng/ml)
blank 0 0.1 1 10 50 100
E
70 kDa Dextran
TNF-α+IFN-γ (ng/ml)
blank 0 0.1 1 10 50 100
F
Viability (%)
Gemcitabine (µM)
G
P app (%)
Gemcitabine (µM)

Figure S1. Response of endothelial barrier made of HUVEC cells to (TNF-α+IFN-g)-induced inflammation. Related to Figure 1. (A) Modeling of flow rate and shear stress changes depending on angle set on the rocker and spatial distribution of shear stress values at 10-degree angle. Simulation was done using finite element analysis in COMSOL Multiphysics. **(B)** Assessment of Dextran permeability with time through bare membrane. Measurements were done at 30, 60 and 120 min, and permeability was plotted in log scale for better visualization. **(C)** VCAM1 (green) and VE-cadherin (red) staining of HUVEC endothelial barrier. Cells were treated with 0-100 ng/mL TNF-α alone or TNF-α+IFN-γ mixture for 48 hours, imaged, and analyzed for expression levels. DAPI was used as a nuclear stain. Scale bar = 50 µm. **(D, E)** Permeability of HUVEC endothelial monolayers in microfluidic channels before and after (TNF-α+IFN-γ)-induced inflammation. Cells were incubated with TNF-α and IFN-γ at concentrations from 0 to 100 ng/mL for 24 hours, and permeability was assessed using 4 kDa (B) and 70 kDa (C) dextran. Sample size = 3 for each condition, data shown as mean ± SD. **(F, G)** Viability of endothelial cells (HUVECs) cultured in microfluidic channel (E) and permeability (P_{app} in %) of endothelial barrier (F) after 3-day treatment with Gemcitabine at varying concentrations. Cell viability was calculated based on a fluorescence signal from live and dead cells and presented as live/dead ratio normalized to control condition without added drug. Barrier permeability was assessed using 70 kDa Dextran and shown in relation to permeability in the absence of the drug. Data shown as mean ± SD (3-4 replicas).

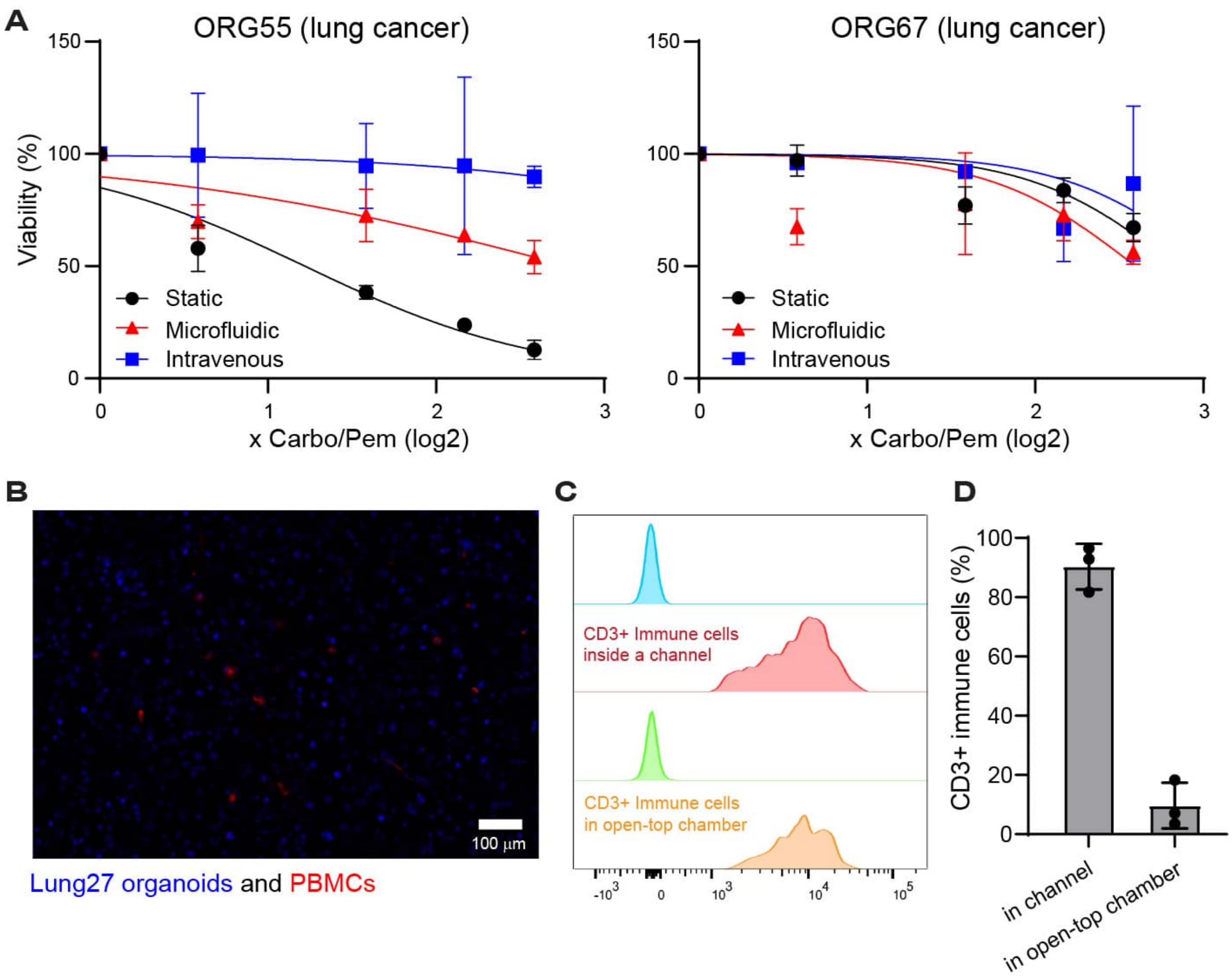

Figure S2. Assessment of drug responses and immune cell infiltration in microfluidic TOC. Related to Figures 2 and 3. (A) IC50 drug response curves for ORG55 and ORG67 cultured in a 384-well plate ("Static") or open-top chambers of TOC ("Microfluidic"). Chemotherapeutics (Carboplatin/Pemetrexed) were added to cells in either setup, and viability was assessed using CellTiter-Glo Luminescent Cell Viability assay. **(B)** Fluorescence image of open-top chamber with Lung27 organoids (blue) and infiltrated PBMCs (red). Organoids and PBMCs were pre-labeled with live stains. Magnification 10x, scale bar = 100 µm. **(C, D)** Quantification of infiltrated PBMCs in the absence of virus infection using flow cytometry. Non-infected organoids and PBMCs were stained with EpCam and CD3 antibodies, respectively. Only CD3+ lymphocytes were quantified. The average percentage of cells in each compartment is shown as mean ± SD (n=3).

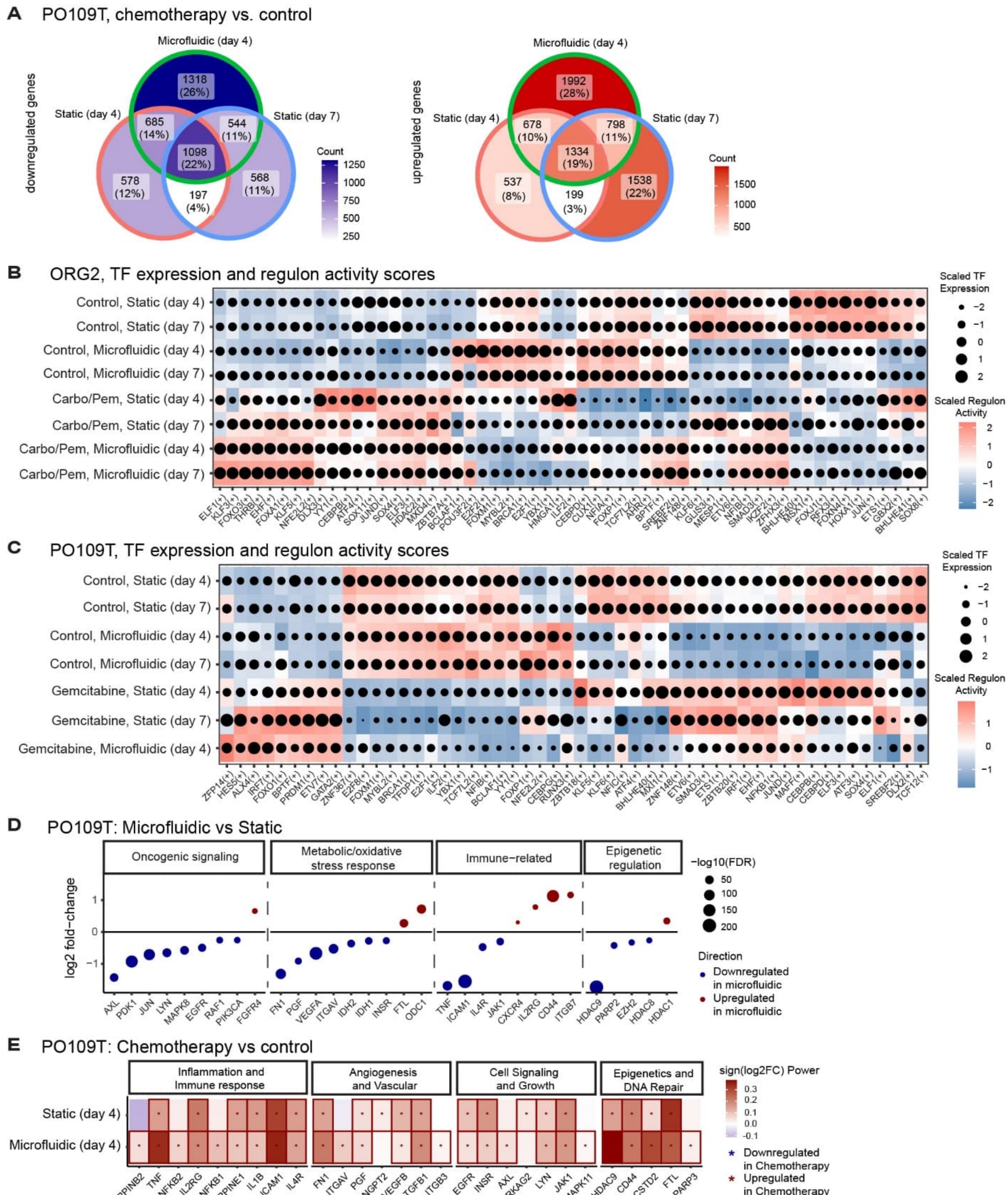

Figure S3. Effect of cancer chemotherapeutics on overall gene and transcriptional factor expression activity. Related to Figures 6 and 7. (A) Schematic showing total gene numbers (common and unique) upregulated (red scale) or downregulated (blue scale) in Microfluidic vs Static conditions, with or without drug treatment, for PO109T cancer models. **(B, C)** Comparison of transcription factor (TF) expression and regulon activity scores for ORG2 (B) and PO109T (C) models under different culturing conditions and drug treatments. Color coding indicates increased (red) or decreased (blue) regulon activity, with black circle

size representing TF expression intensity. No data is shown for PO109T on day 7 in microfluidic settings due to low cell recovery. (**D**) Differential expression of genes in oncogenic, metabolic/oxidative stress response, immune-related, and epigenetic regulation pathways between Microfluidic and Static conditions for PO109T. Red and blue dots represent upregulated and downregulated genes in Microfluidic conditions, respectively, with spot size indicating FDR. (**E**) Chemotherapeutic treatments cause different gene expression changes in Static vs Microfluidic conditions for PO109T, with significant differences highlighted in square frames.
